# *De novo* assembly of highly polymorphic metagenomic data using *in situ* generated reference sequences and a novel BLAST-based assembly pipeline

**DOI:** 10.1186/s12859-017-1630-z

**Published:** 2017-04-26

**Authors:** You-Yu Lin, Chia-Hung Hsieh, Jiun-Hong Chen, Xuemei Lu, Jia-Horng Kao, Pei-Jer Chen, Ding-Shinn Chen, Hurng-Yi Wang

**Affiliations:** 1 Department of Life Science, National Taiwan University, Taipei, 106 Taiwan; 2 Department of Forestry and Nature Conservation, Chinese Culture University, Taipei, 111 Taiwan; 3 Laboratory of Disease Genomics and Individualized Medicine, Beijing Institute of Genomics, the Chinese Academy of Sciences, Beijing, 100101 China; 4 Graduate Institute of Clinical Medicine, National Taiwan University, Taipei, 100 Taiwan; 5 Genomics Research Center, Academia Sinica, Taipei, 115 Taiwan; 6 Institute of Ecology and Evolutionary Biology, National Taiwan University, Taipei, 106 Taiwan; 7 Research Center for Developmental Biology and Regenerative Medicine, National Taiwan University, Taipei, 100 Taiwan

**Keywords:** Next generation sequencing, Metagenomics, Hepatitis B virus, Sequence assembly, Assembly pipeline

## Abstract

**Background:**

The accuracy of metagenomic assembly is usually compromised by high levels of polymorphism due to divergent reads from the same genomic region recognized as different loci when sequenced and assembled together. A viral quasispecies is a group of abundant and diversified genetically related viruses found in a single carrier. Current mainstream assembly methods, such as Velvet and SOAPdenovo, were not originally intended for the assembly of such metagenomics data, and therefore demands for new methods to provide accurate and informative assembly results for metagenomic data.

**Results:**

In this study, we present a hybrid method for assembling highly polymorphic data combining the partial *de novo*-reference assembly (PDR) strategy and the BLAST-based assembly pipeline (BBAP). The PDR strategy generates *in situ* reference sequences through *de novo* assembly of a randomly extracted partial data set which is subsequently used for the reference assembly for the full data set. BBAP employs a greedy algorithm to assemble polymorphic reads. We used 12 hepatitis B virus quasispecies NGS data sets from a previous study to assess and compare the performance of both PDR and BBAP. Analyses suggest the high polymorphism of a full metagenomic data set leads to fragmentized *de novo* assembly results, whereas the biased or limited representation of external reference sequences included fewer reads into the assembly with lower assembly accuracy and variation sensitivity. In comparison, the PDR generated *in situ* reference sequence incorporated more reads into the final PDR assembly of the full metagenomics data set along with greater accuracy and higher variation sensitivity. BBAP assembly results also suggest higher assembly efficiency and accuracy compared to other assembly methods. Additionally, BBAP assembly recovered HBV structural variants that were not observed amongst assembly results of other methods. Together, PDR/BBAP assembly results were significantly better than other compared methods.

**Conclusions:**

Both PDR and BBAP independently increased the assembly efficiency and accuracy of highly polymorphic data, and assembly performances were further improved when used together. BBAP also provides nucleotide frequency information. Together, PDR and BBAP provide powerful tools for metagenomic data studies.

**Electronic supplementary material:**

The online version of this article (doi:10.1186/s12859-017-1630-z) contains supplementary material, which is available to authorized users.

## Background

Next-generation sequencing (NGS) has become the mainstream method for obtaining high quantities of genomic data during the past decade, and the increased accessibility of massive datasets has driven up the need for compatible analytic algorithms and software [1]. There are several key components for an assembly algorithm, including the capacity to handle massive data sets, the accuracy and efficiency of the assembly, the nature of the data set itself, and the intended use of the assembly results. The former two are dependent of the hardware and algorithms implemented, whereas the latter two influences the optimization strategy and the type of information to be extracted during assembly. For example, metagenomic studies commonly aim to understand the composition and relative abundances of the data set as well as the intra-species or inter-population heterogeneity, therefore the assembly depth and length as well as accuracy are prioritized for such data sets [2].

A viral quasispecies is a group of highly genetically related viruses found in a single carrier and can be both abundant (viral titer ≈ 10^6^-10^9^ ge/ml) and greatly diversified (nucleotide diversity ≈ 10^−2^-10^−3^) within patient carriers [3–5]. Two main NGS platforms, 454/Roche pyrosequencing [6] and Illumina Genome Analyzer [7], have been commonly used for recent quasispecies-related studies. Pyrosequencing has longer sequence reads and typically does not require data set assembly [8–10], although some studies still performed *de novo* assembly [11] or reference sequence assembly [12, 13]. Illumina sequencing generates much larger data sets compared to pyrosequencing, but its shorter read length limits the efficiency for *de novo* assembly [2]. Therefore, Illumina sequenced viral quasispecies data sets are usually assembled using reference sequences as templates [14–17] while *de novo* assembly is applicable but not commonly used [18].

The high throughput Illumina platform, compared to the pyrosequencing platform, is capable of detecting greater amounts of genetic variation within viral quasispecies [15]. However, a major challenge for Illumina quasispecies NGS studies is the sequence assembly of the data sets. Sequence assembly using a reference approach is not only subject to bias of the chosen reference sequence, but also assembles less reads and thus less genetic variation information in the assembly [15]. *De novo* assembly should be able to provide the most complete and accurate genetic information of NGS data, but can be hindered by regions with high levels of diversity. The commonly used *de novo* assembly algorithms, such as Velvet [19], SOAPdenovo [20], CLC Genomics Workbench (CLC, CLC bio, Aarhus, Denmark), and Euler-SR [21], were not originally intended for the assembly of metagenomics data with high diversity and coverage depth. Recent progress have been made in the development of *de novo* assembly algorithms for metagenomes, such as MetaVelvet [22] and Genovo [23].

In this study, we propose a partial *de novo*-reference assembly strategy, PDR, which is a *de novo*-reference hybrid assembly strategy that utilizes the completeness of *de novo* assembly while complementing its low-efficiency with reference assembly. PDR generates an *in situ* reference sequence by *de novo* assembly of a smaller yet less diverse partial data set followed by the reference assembly of the full data set. Results show that the PDR assembly results are more complete and accurate than direct *de novo* or reference assembly of highly polymorphic metagenomic data sets. We also present a novel BLAST-based assembly pipeline, BBAP, capable of both *de novo* and reference assembly specifically designed for assembly of metagenomic data sets. The assembly efficiency and accuracy of both PDR and BBAP were examined using actual NGS data sets as well as *in silico* generated simulated NGS data sets and compared with the assembly results of other assembly methods.

## Results

To examine the performance of BBAP and the proposed hybrid assembly strategy, we acquired 12 NGS data sets of HBV viral quasispecies from 7 HBV patient samples [24]. The 12 data sets used for assembly consisted of an average of 21,494,295 101-bp raw reads (RRs), 14,388,844 high quality reads (HQRs, quality score ≧ 20 for all bases; i.e., sequencing error rate = 1%), and 60,228 HRURs (high redundancy unique representative reads; unique representative reads with redundancy ≧ 5, Table 1 and Additional file 1: Table S1). The optimized parameters for BBAP assembly are listed in Additional file 1: Table S2. The same parameters were used for all BBAP assemblies in this study unless mentioned otherwise.

**Table 1:** Average assembly statistics of all 12 data sets using BBAP with multiple approaches

	PD^a^	FD^b^	SR^c^	PDR^d^
RRs	214,942	21,494,295	21,494,295	21,494,295
HQRs	143,912	14,388,844	14,388,844	14,388,844
URs	27,150	860,144	860,144	860,144
HRURs	6264	60,228	60,228	60,228
RiHRURs	116,555	13,388,423	13,388,423	13,388,423
Contigs assembled^e^	2.1	46.0	1.0	3.9
Max contig length	3119	1473	3,207	3148
Average contig length	2319	321	3207	1268
% of Mapped HRURs	95.9%	70.3%	67.4%	69.9%
% of Mapped RiHRURs	80.4%	68.7%	82.7%	84.5%

The full data sets were used in the BBAP assembly with FD, SR, and PDR approaches, whereas partial data sets consisting of 1% of randomly selected RRs were used in the BBAP PD assembly approach

^a^Partial data set *de novo* assembly

^b^Full data set *de novo* assembly

^c^Sanger reference assembly

^d^Partial data set reference assembly of the full data set

^e^Only minimum assembled contig length > 150 bp was shown

*RRs* raw reads, *HQRs* high quality reads (quality score threshold = 20, i.e., sequencing error rate = 1%), *URs* unique representative reads, *HRURs* high redundancy unique representative reads (unique representative reads with redundancy threshold = 5), *RiHRURs* reads included in high redundancy unique representative reads

### BBAP *de novo* assembly of full and partial data sets

**Fig. 1:**
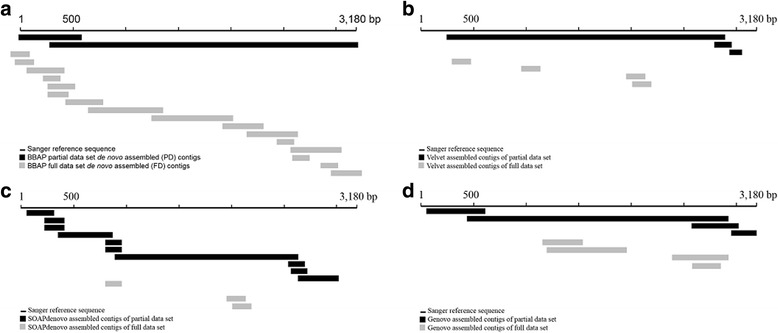
Assembly results of full and partial D2_1 data set by **a** BBAP, **b** Velvet, **c** SOAPdenovo, and **d** Genovo. The contigs were aligned to the Sanger reference sequence. MetaVelvet assembly results for both full and partial D2_1 data set were identical to those of Velvet and thus not shown

The *de novo* assembly of the full data sets (FD) resulted in an average of 46.0 contigs (minimum length of 150 bp) for each library with an average contig length of 321 bp, suggesting that the assembly results were fragmentized (Table 1 and Additional file 1: Table S3). For *de novo* assembly of partial data sets (PD) of each data set, five partial data sets were initially randomly generated and assembled independently. Because the PD assembly results of the partial data sets from each library were highly similar (data not shown), a single partial data set and its assembly results were used for representation of the sample in further analyses. The PD assembly yielded fewer number of contigs and longer average maximum contig lengths, indicating the PD assembly results were not as fragmentized as FD assembly. Furthermore, PD assembly required fewer contigs than the FD assembly to span the full genome to recover the full length HBV genome (Fig. 1a, Additional file 2: Figure S1). PD assembly also yielded a higher proportion of mapped HRURs (95.9% vs 70.3%) and RiHRURs (reads included in high redundancy unique representative reads, 80.4% vs. 68.7%) than FD, further demonstrating its better assembly efficiency.

Fragmentation is possibly due to high polymorphic reads from the same genomic regions recognized by BBAP as different haplotypes and subsequently assembled into separate clusters. The proportion of polymorphic sites in overlapping contig regions of D2_1 FD assembly was 10 times higher than that in non-overlapping regions (0.238 vs. 0.022; *p* < 10^−10^). A similar trend was also found in D2_1 PD assembly (Additional file 1: Table S4). The shorter FD assembled contigs (<300 bp) had a significantly higher proportion of polymorphic sites than the longer FD assembled contigs (Additional file 2: Figure S2, Student’s *t*-test, *p* < 0.05). HRURs that were included or excluded in the partial data sets (for PD assembly) had average redundancies of 1,808X (n = 75,173) and 38X (n = 647,561), respectively, within the full data set. Additionally, the redundancies of the included HRURs in the full and partial data sets were highly correlated (R^2^ = 0.9997). This suggests the random selection partial data sets was unbiased and effectively excluded HRURs of low redundancies, resulting in lower polymorphism levels and, in turn, less fragmented assembly results.

### BBAP reference assembly with different reference sequences

To fully represent the full data set, the PD assembled contigs were used as references for the reference assembly of the full data set (PDR). For comparison purposes, a Sanger sequence from each patient sample was chosen as the reference sequence for the reference assembly of the full data set (SR). SR assembly resulted in single contigs with average lengths of 3207 bp, whereas PDR assembly produced an average of 3.9 contigs with maximum and average lengths of 3148 bp and 1268 bp, respectively (Table 1 and Additional file 1: Table S3). Both PDR and SR recovered full HBV genomes and similar levels of polymorphism in the consensus sequences (Additional file 1: Table S5), but the PDR assembly additionally identified HBV structural variants (Additional file 1: Table S6, Additional file 2: Figure S3-S5 and Additional file 3: SA).

PDR alignment accuracy was also higher than SR. SR assembly of D2_1 resulted in a single contig with 50,587 HRURs, but only 50,211 of the SR assembled HRURs were mapped to the two main PDR assembled contigs (M1 and M2; Additional file 2: Figure S6, 50,396 HRURs) covering the full HBV genome and have identical sequences as the SR contig. Not only did the remaining 376 HRURs all mapped to one of the nine PDR assembled variant contigs, but the SR alignment qualities of those 376 HRURs was less optimal than the 50,211 HRURs, shown by the significantly greater BLAST e-value and lower BLAST alignment score (Wilcoxon rank-sum test, *p* < 0.001), both supporting the higher alignment accuracy of PDR assembly. Overall, results of SR assembly and PDR assembly were similar in recovering sequence variation, but the latter included more HRURs and RiHRURs with increased accuracy due to the additional mapping options of the shorter HBV variant contigs provided by the *de novo* assembly of the partial data set, whereas the lower assembly accuracy of the former resulted in low quality alignments and slightly more polymorphic sites.

We were able to measure the polymorphism level of BBAP assembly results (Additional file 2: Figure S6) by calculating the nucleotide frequencies for each position (Additional file 1: Table S7, Additional file 2: Figure S7 and Additional file 3: SB). Furthermore, the nucleotide frequencies derived from BBAP PDR assembly were validated by pyrosequencing (Additional file 1: Table S8), demonstrating the assembly results of BBAP are reliable.

### BBAP assembly results compared with other assembly methods

We next compared the efficiency and accuracy of BBAP to different assembly methods using both full and partial D2_1 data set. Similar to BBAP FD, the full data set assemblies by Velvet, MetaVelvet, SOAPdenovo, and Genovo resulted in fragmented contigs. *De novo* assembly of full data set with Velvet resulted in 13 contigs with maximum and average lengths of 1102 bp and 303 bp, respectively (Table 2), and recovered only 19% of the HBV genome (Fig. 1b, Additional file 2: Figure S1). MetaVelvet assembly results, which are based on initial Velvet assembly results, did not show any improvement and were completely identical to Velvet assembly results for both full and partial data set. SOAPdenovo generated 8 assembled contigs with maximum and average lengths of 934 bp and 340 bp, respectively, and covered 14% of the HBV genome (Fig. 1c). Genovo assembly for the D2_1 data set resulted in a total of 60 contigs with maximum and average contig lengths of 1352 bp and 395 bp, respectively, but only 44% of the HBV genome were recovered (Fig. 1d, Additional file 2: Figure S1).

**Table 2:** Comparison of D2_1 assembly results with different methods and different data set sizes

	Max length	Average length	Number of contigs	% of HBV genome recovered	Contigs that map to reference HBV genome	Contigs with HBV structural variants
BBAP/FD	998	263	52	100%	16	30
Velvet/Full	1102	303	13	19%	4	0
MetaVelvet/Full	1102	303	13	19%	4	0
SOAPdenovo/Full	934	340	8	14%	3	0
Genovo/Full	1352	395	60	44%	4	34
BBAP/PD	2924	692	6	100%	3	3
Velvet/Partial	2576	973	3	89%	3	0
MetaVelvet/Partial	2576	973	3	89%	3	0
SOAPdenovo/Partial	1723	390	10	95%	10	0
Genovo/Partial	2427	481	12	91%	4	7

We proposed that the high polymorphic nature of virus quasispecies may have hindered the efficiency of sequence assembly, and a randomly extracted yet less polymorphic partial data set may provide a better start for initial assembly as shown in FD vs. PD assemblies. Assembly results of different methods all show that the assembly of the partial data set not only generated longer contigs, but also recovered more than 90% of the full HBV genome, demonstrating that exclusion of low redundant HRURs by random selection of partial data effectively reduced level of polymorphism which, in turn, improved the assembly results as judged by contig length and coverage (Table 2, Additional file 2: Figure S1).

**Fig. 2:**
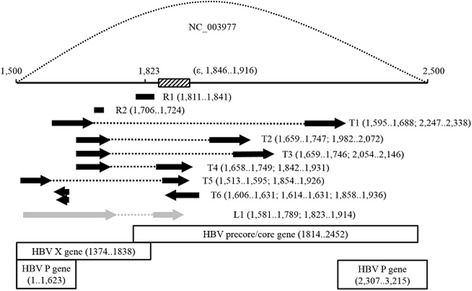
Schematic summary of corresponding HBV genome (NC_003977) regions for assembled contigs identified as HBV variants. Arrows indicate 5’ to 3’ direction. Only reads containing the sequences spanning the junction regions were assembled separately into variant contigs; reads spanning non-junction regions of the variant contigs (dotted lines) were assembled into the main HBV contig. The L1 sequence, which is similar to T5, resulted from HBV variant validation with PCR using specialized primers followed by Sanger sequencing. Positions are in correspondence with NC_003977, with dotted lines representing the remaining portion of the circular HBV genome, and the boxed section indicating the encapsidation signal (or episilon, ε)

We also noticed that BBAP had better performance in recovering structural variants than the other methods tested. While some of BBAP assembled HBV variants were validated by PCR sequencing (Fig. 2), both Velvet/MetaVelvet and SOAPdenovo did not identify any contigs with HBV structural variation. Although Genovo assembled 34 structural containing contigs, their accuracies were questionable as most of them with non-retraceable junction regions (Additional file 2: Figure S8 and Additional file 3: SC).

### Results of *in silico* data set assembly

For a more general assessment and comparison of BBAP performance, *in silico* NGS data sets were generated from the NCBI HBV complete genome and assembled separately using BBAP FD, Velvet, MetaVelvet, SOAPdenovo, and Genovo. Data set sizes were set to 1,726,462 (55,799X), 172,646 (5,579X), 17,264 (557X), and 1726 (55X) HQRs in combination with error rates of 10^−2^, 10^−3^, and 10^−4^/site. Due to computing time considerations, the maximum simulated data set size of 55,799X was approximately 10% of the D2_1 data set size. Five independent data sets were generated for each parameter combination. BBAP assembly results were highly consistent regardless of the data set parameter values. All but one of the 60 assembly results had both perfect coverage and accuracy; the lone standout assembly result had perfect coverage but a 0.9996 (3214/3215) accuracy (Table 3 and Additional file 1: Table S9). The single “inaccurate” nucleotide was not an assembly error, but rather a degenerate nucleotide (Y) representing the reference nucleotide (T, 2/3 or 0.67) and the *in silico* generated erroneous nucleotide (C, 1/3 or 0.33). The corresponding *in silico* data set was generated with the highest error rate (0.01) and smallest data set size (55X), which is the most likely parameter value combination for erroneous nucleotides to exceed the minimum nucleotide frequency threshold (0.2).

**Table 3:** Assembled results of in silico generated data sets from the reference HBV genome by different methods^a^

Data set size	Method	BBAP FD			Velvet			SOAPdenovo			Genovo		
	Error rate	10^−4^	10^−3^	10^−2^	10^−4^	10^−3^	10^−2^	10^−4^	10^−3^	10^−2^	10^−4^	10^−3^	10^−2^
55X	Coverage	1	1	1	1	1	1	**0**	**0**	1	1	1	1
	Accuracy	1	1	0.99	1	1	1	**0**	**0**	1	1	1	1
	# of contigs	1	1	2	1	1	1	**0**	**0**	1	1	1	1
557X	Coverage	1	1	1	1	0.99	1	**0**	1	**0.27**	1	1	1
	Accuracy	1	1	1	1	1	0.99	**0**	0.99	**0.99**	1	1	1
	# of contigs	1	1	2	1	1	9	**0**	1	**5**	1	1	1
5,579X	Coverage	1	1	1	0.99	0.96	**0.03**	1	**0.01**	**0.43**	1	1	1
	Accuracy	1	1	1	1	1	**0.59**	1	**0.20**	**0.99**	1	1	0.99
	# of contigs	1	1	1	1	6	**1**	1	**0**	**11**	1	1	1
55,799X	Coverage	1	1	1	0.98	**0**	**0.11**	**0.02**	**0**	**0.04**	1	1	1
	Accuracy	1	1	1	1	**0**	**0.97**	**0.40**	**0**	**0.80**	1	1	0.99
	# of contigs	1	1	1	3	**0**	**2**	**0**	**0**	**1**	3	2	5

^a^Results represent averages of the assembly results of 5 replicate data sets. Bold areas indicate average assembly results with <80% coverage

Velvet assembly of the *in silico* data sets produced mixed results (Additional file 1: Table S10). Data sets with low error rates and/or small data set sizes were assembled with near perfect coverage and accuracy, whereas both large data sets and high error rates were poorly assembled. As the degree and amount of polymorphism are proportional to the error rate and data set size, respectively, results suggest Velvet is inefficient in assembling highly polymorphic data sets. Unlike the assembly results for D2_1 data sets, MetaVelvet *in silico* data set assembly results, compared to Velvet results, were improved with higher coverage and less fragmentation (Additional file 1: Table S11). MetaVelvet has wider parameter handling range than Velvet, but was still unable to assemble highly polymorphic data sets with high error rates and large data set sizes. Similar to that of Velvet and MetaVelvet, SOAPdenovo could not efficiently assemble data sets of high polymorphism (large data set size and high error rate). In addition, SOAPdenovo also performed poorly when assembling data sets of low polymorphism (low error rate and small data set size). Only data sets of medium sizes and error rates were efficiently assembled by SOAPdenovo (Additional file 1: Table S12). Genovo assembly of smaller data set sizes (55X, 557X, and 5,579X), regardless of the error rate, were highly consistent, with only a single nucleotide assembly error among all 45 assembly results (Additional file 1: Table S13). The assembly result for the largest data sets (55,799X) were slightly fragmentized across all error rates and on average 4 assembly errors were identified among high error rate (0.01) data sets.

## Discussion

We developed BBAP, an assembly pipeline designed for the accurate and efficient assembly of highly polymorphic metagenomic NGS data sets. BBAP implements a unique BLAST-based greedy algorithm to assemble data set reads and provides multiple intuitive parameters, depending on the nature of the data set, the sequencing platform, and information demands, to adjust the threshold for read alignment, variant retention, and error removal during assembly. BBAP assembly results of both real and simulated NGS data sets were of higher quality than assembly results of other methods compared.

We also introduce a new partial *de novo*-reference (PDR) assembly strategy, which *in situ* generates reference sequences by *de novo* assembly of a randomly extracted partial data set to be subsequently used for the reference assembly of the full data set. Current assembly approaches typically assemble the full data set straightforward with either *de novo* or reference assembly methods, each with their respective advantages and disadvantages. Reference assembly is a much more direct process than *de novo* assembly which reduces alignment ambiguities and low coverage issues. However, the quality of reference assembly is reliant on the representation level of the reference sequence, as the assembly result will be biased towards the reference sequence and sequence variations not represented by the reference sequence will not be captured. *De novo* assembly, which is independent of reference sequences, possesses the potential to generate a more complete assembly result including majority consensus sequences and minor variant sequences, but can be hindered by coverage gaps that lack sequencing information and polymorphic regions with high levels of diversity as shown in Tables 1 and 2.

The partial *de novo*-reference assembly strategy utilizes the advantages of both traditional approaches to contemplate each other. *De novo* assembly of a randomly extracted yet less polymorphic partial data set provides assembly results that are more complete and highly representative of both majority sequence as well as minor variant sequences in the full data set. In turn, the following reference assembly not only assembles more reads due to the accurate representation of the reference sequences, but also has increased assembly accuracy than both straight-up *de novo* and reference assemblies (Table 1). More importantly, the improved quality of assembly resulting from this hybrid PDR approach was not limited to BBAP, as better assembly results using partial data sets were also demonstrated by Velvet, MetaVelvet, SOAPdenovo, and Genovo (Table 2).

The assembly efficiency of metagenomics data sets is also dependent on the algorithms each assembly method employs. Velvet, MetaVelvet, and SOAPdenovo all assemble NGS data sets through the construction of de Bruijn graphs and Eulerian paths. De Bruijn graphs contain overlapping sequence information represented by branching nodes and stemming vertices, and is extremely sensitive and results quickly deteriorate even with the slightest amount of polymorphism [21]. The assembly algorithm of Velvet and SOAPdenovo both manipulate the constructed de Bruijn graph with error removal and simplification to generate optimal assembly results, which effectively excludes the essential polymorphism information vital to metagenomics data sets during assembly. In contrast, MetaVelvet decomposes the de Bruijn graphs into individual subgraphs and assembles each subgraphs into separate contigs. On the other hand, BBAP adopts a greedy assembly approach by incorporating and clustering sequence reads through BLAST results, and Genovo implements a Bayesian-based probabilistic model and takes into account the potential presence of multiple genomes in the data set. Therefore, it was reasonably expected for BBAP, MetaVelvet, and Genovo to have better assembly results than Velvet and SOAPdenovo when assembling metagenomics data sets, and this was consistent with our results that support BBAP, MetaVelvet, and Genovo are better equipped to assemble metagenomics data sets than Velvet or SOAPdenovo.

We compared the average assembly times for *in silico* and NGS data sets on our server (E5310 1.6GHz x4 *x*2, 12GB RAM) between all methods to further assess the performance of both BBAP and PDR. For smaller *in silico* data sets (data set size ≦5,579X or 17.44 Mb) BBAP assembly time was slightly longer than Velvet, MetaVelvet, and SOAPdenovo, but still within a couple minutes (Additional file 1: Table S14). BBAP assembly time for the largest *in silico* data sets tested (data set size = 55,799X or 174 Mb) were similar to the assembly time by the other methods except Genovo, which required considerably much more assembly time than BBAP or the other methods for all *in silico* data sets. The average BBAP PDR assembly time (624 s) for the 12 NGS data sets was drastically faster than the average BBAP FD assembly time (14,347 s). Overall, results suggest not only do both BBAP and PDR individually increase assembly efficiency and accuracy compared to their respective counterparts, but the combination of BBAP and PDR together further improves the overall assembly quality of metagenomic data sets.

Viral pathogens are responsible for the majority of pandemic and epidemic diseases listed by the World Health Organization. Recent studies have utilized the advantages of NGS data sets of the viral quasispecies genome to construct genome-wide diversity profiles for studying the virus-host interactions during infection and, treatment and vaccination [8, 10, 11, 15, 17]. Resistance associated variants and novel variants of the viral quasispecies usually are rare and not detectable by conventional or low depth sequencing, therefore detection of minor variants is clinically important for customizing patient management and treatment strategies [10, 16]. Our results show that BBAP and PDR not only provided an accurate assembly sequence but also generates a high resolution diversity profile of the data set. Additionally, we were able to detect and recover novel variants that were otherwise undetectable to alternative assembly methods.

## Conclusions

Assembly of a highly polymorphic NGS data set is a complicated process as it involves multiple steps (such as quality control, read assembly and error removal) and is dependent of several prerequisite factors (data set type, sequencing platform, intended use of results, etc.). In addition, a functional understanding of the algorithms and sufficient parameters are important for the optimization of assembly results. We believe both BBAP and the partial *de novo*-reference assembly strategy will provide a powerful tool for future metagenomic and viral quasispecies studies.

## Methods

### BLAST-based assembly pipeline

**Fig. 3:**
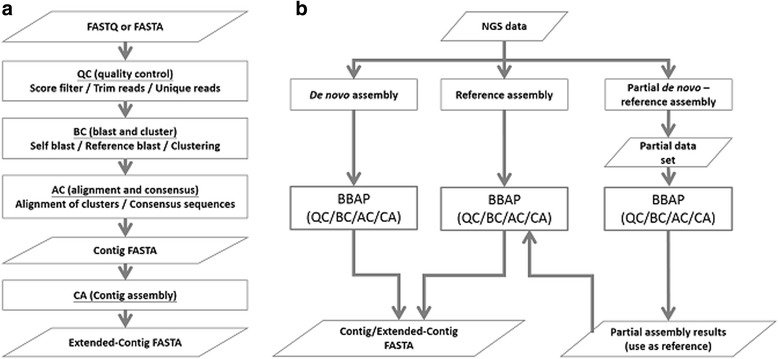
Flow chart summary of BBAP. **a** The pipeline is divided into four major steps: quality control (QC), blast and cluster (BC), alignment and consensus determination (AC), and contig assembly (CA). **b** BBAP can perform *de novo* assembly, reference assembly, or partial *de novo*-reference assembly, which includes the initial *de novo* assembly of a randomly extracted partial data set followed by the reference assembly of the full data set using the assembly results from the initial assembly as reference sequences

The BLAST-based assembly pipeline, BBAP, is divided into four major steps: quality control (QC), blast and cluster (BC), alignment and consensus determination (AC), and contig assembly (CA) (Fig. 3a). BBAP assembles high quality sequences into contigs according to BLAST results. Alignment files of the assembled contigs are generated as a result. The contigs are further assembled into extended-contigs and resulting in contig sequences, a log file, and a statistical analysis of the assembly. All steps, with the sole exception of BLAST, used in-house developed perl scripts.

The QC step excludes sequences with low quality scores, trims sequences from both ends, removes redundant identical sequences, and filters unique representative sequences with low redundancy. First, raw reads (RRs) that include any called base with a quality score less than the given threshold is omitted. The remaining high quality reads (HQRs) are trimmed from both ends for the given length to remove barcodes, artificial sequences such as linker, adapters or vectors, and error-prone regions that are more frequently found in the terminal regions for some sequencing platforms. Identical HQRs are compressed and represented by a single unique representative read (UR) while retaining the redundancy count information. Unique representative reads with redundancy counts greater than or equal to the given threshold, high redundancy unique representative reads (HRURs), are retained for further assembly.

For *de novo* assembly, the BLAST and cluster step (BC) is initiated with the reciprocal BLAST of the HRURs fasta file. The BLAST parameter of repeat masking was set to include repetitive regions into the results (-F “”). BLAST results with gaps or e-value, identity, or BLAST length not meeting the given thresholds were excluded from further assembly. During clustering, if two reads are BLASTed to one another and are both unassigned, then they are assigned to a same new cluster. If only one read has been assigned a cluster, then the unassigned read is added to the cluster of the assigned read. If both have been separately assigned to different clusters, then the two clusters are merged into one single cluster. Finally, clusters with number of assigned reads less than the given threshold sequence number are excluded from further assembly.

The BC step of reference assembly is similar to that of *de novo* assembly but with some minor differences. Instead of reciprocal BLAST, the HRURs fasta file is BLASTed to the reference sequences. If a read has identical e-values for multiple reference sequences, the read will be assigned to the reference sequence with the longest sequence length.

The alignment and consensus determination step (AC) calculates the alignment position for each read of a cluster based on its BLAST results. Only top BLAST results with identity and BLAST length greater than the given thresholds were used for alignment. Consensus sequences were calculated for each base according to the alignment results. Nucleotides with frequencies greater than or equal to the given threshold are retained for polymorphic sites.

Contigs with identical terminal sequences longer than the given threshold are merged together into extended-contigs. Identical terminal sequences were identified by self-BLAST of contigs. This step is optional and dependent on the nature of the data set.

Overall, BBAP uses BLAST results (reciprocal BLAST for *de novo* assembly, and data set to reference sequence BLAST for reference assembly) to cluster reads into contig groups to increase computation efficiency of following steps. The reads in each contig group are then positioned/aligned according to their respective BLAST results into contigs. The grouped reads are then extended into contigs according to positioning/alignment information provided from the BLAST results in a greedy strategy manner. Extension of contigs and prevention of assembly artifacts (such as artificial chimeras) are directly dictated by the BLAST identify and length threshold parameters, and indirectly effected by quality control parameters, including the QC-score threshold and the redundancy threshold.

BBAP can assemble data sets with or without a reference sequence by reference assembly or *de novo* assembly, respectively. We also introduce a third assembly strategy, the partial *de novo-*reference assembly approach (Fig. 3b). A randomly extracted partial data set is first *de novo* assembled, and then the resulting contig sequences are used as reference sequences to assemble the entire data set through reference assembly.

### Next generation sequencing data set assembly and statistical analyses

NGS data sets were downloaded from a previous study [24], which consisted of 12 libraries derived from 7 patients chronically infected with HBV within a single family (Additional file 1: Table S15). The full data set was separately assembled with BBAP through full data set *de novo* (FD) assembly, Sanger reference (SR) assembly, and partial *de novo*-reference (PDR) assembly. A single Sanger sequence from each patient sample was chosen and used as the reference sequence for the SR assembly of the corresponding full data set. For the PDR assembly, partial data sets were constructed independently by randomly choosing 1% of the RRs from the full data set and assembled *de novo*, and the results of the partial data set *de novo* (PD) assembly were used as reference sequences for the reference assembly of the full data set. Partial data sets of different ratios were assembled and 1% partial data sets generated the most optimal assembly results (Additional file 1: Table S16 and Additional file 3: SD). Assembly results of different BBAP methods were then compared to each other.

Variant contigs were identified by BLAST against the NCBI HBV complete genome sequence (NC_003977), the Sanger reference sequence, and the NCBI nr/nt database. To verify that the identified variants were not artifacts of incorrect assembly by BBAP, sequences of at least 20 bp and spanning the junction regions of the structural variations were searched for in both the RRs and HQRs fasta files.

The full data set and partial data sets of one library, D2_1 (Additional file 1: Table S15), were also assembled using all methods. Statistical analyses and comparisons between assembly methods were performed with perl scripts.

### *In silico* data set assembly

We also compared the performance of different assembly methods by using simulated data sets. *In silico* data sets were generated by randomly generating 101 bp reads from the reference NCBI HBV complete genome, NC_003977. To mimic observed polymorphism from virus diversity or sequencing error of NGS, different error rates, 10^−2^, 10^−3^, and 10^−4^/site, were applied to the simulated reads. Data set sizes were set to 1,726,462 (55,799X), 172,646 (5,579X), 17,264 (557X), and 1726 (55X) HQRs. Five independent data sets were generated for each parameter combination, error rate and dataset size. Data sets were assembled using BBAP FD assembly, Velvet, MetaVelvet, SOAPdenovo, and Genovo. All *in silico* data sets, except for data sets of high error rate (0.01) coupled with small data set sizes (55X and 557X), used the same BBAP parameter values for NGS *de novo* assembly. For the high error rate-low coverage depth data sets, the redundancy threshold was reduced from 5 to 1 to compensate for its low redundancy. For Velvet, MetaVelvet, and SOAPdenovo assembly, the k-mer size was optimally set to 57, 57, and 63, respectively. For Genovo assembly, different numbers of iterations were used for data sets of different coverage depths because of the extreme long run time for larger data sets; the number of iterations for data sets with coverage depths of 55,799X, 5,579X, 557X and 55X was 10, 2000, 10,000, and 10,000, respectively.

## Additional files

Additional file 1: Table S1. Statistics of next generation sequencing data set of HBV genome from patient serum. **Table S2**: Parameters used for *de novo*, reference, and partial *de novo* reference BBAP assembly. **Table S3**: Assembly results of individual data sets using BBAP with multiple approaches. **Table S4**: Comparison of polymorphism between non-overlapping and overlapping regions of D2_1 assembled contigs alignment. **Table S5**: Comparison of polymorphism levels between assembly results of BBAP PDR and SR assemblies. **Table S6**: Summary of assembled contigs from the PDR assembly of D2_1 NGS data set. **Table S7**: Top ten non-synonymous frequency positions of the HBV quasispecies. **Table S8**: Nucleotide frequencies derived from BBAP PDR assembly and pyrosequencing. **Table S9**: Results of BBAP *de novo* assembled in silico NCBI HBV complete genome (NC_003977) data sets (*n* = 5). **Table S10**: Results of Velvet assembled *in silico* NCBI HBV complete genome (NC_003977) data sets (*n* = 5). **Table S11**: Results of MetaVelvet assembled *in silico* NCBI HBV complete genome (NC_003977) data sets (*n* = 5). Table S12: Results of SOAPdenovo assembled *in silico* NCBI HBV complete genome (NC_003977) data sets (*n* = 5). **Table S13**: Results of Genovo assembled *in silico* NCBI HBV complete genome (NC_003977) data sets (*n* = 5). **Table S14**: Assembly time required for *in silico* data sets by BBAP, Velvet, MetaVelvet, SOAPdenovo, and Genovo. **Table S15**: Summary of study subjects and samples. **Table S16**: Summary of assembly results for D2_1 partial data sets of different size ratio. (DOC 461 kb)

Additional file 2: Figure S1. Comparison of HBV recover ratio by BBAP, Velvet, SOAPdenovo, and Genovo assembly of full and partial D2_1 data sets. **Figure S2**: Correlation between assembled scaffold length and scaffold degeneracy for all 12 data sets. **Figure S3**: (a) Nucleotide sequence of the R1 scaffold. (b) Schematic alignment of the R1 scaffold, HBV X gene and HBV precore/core gene. **Figure S4**: Schematic diagram of the T1 scaffold and its corresponding HBV genome regions. **Figure S5**: Schematic diagram of the T6 scaffold and its corresponding HBV genome and Sanger reference sequence regions. **Figure S6**: Alignment of Sanger (SR) and partial D2_1 data set assembled scaffolds reference assembled (PDR) scaffolds to the Sanger reference sequence. **Figure S7**: Diversity profile of D2_1 HBV quasispecies according to assembly results of partial data set reference assembly of the full data set. **Figure S8**: Schematic diagram of two Genovo assembled scaffolds with identified HBV structural variants and its corresponding HBV genome regions. (PDF 489 kb)

Additional file 3: **A**. Variant sequences and human genome sequences. **B**. Diversity profile of D2_1 HBV quasispecies. **C**. Structure variation by Genovo. **D**. Determining optimal size of partial data set. (DOC 67 kb)

## Abbreviations

AC: Alignment and consensus determination
BBAP: BLAST-based assembly pipeline
BC: Blast and cluster
CA: Contig assembly
CLC: CLC Genomics Workbench
FD: *De novo* assembly of the full data set
HQRs: High quality reads
HRURs: High redundancy unique representative reads
NGS: Next generation sequencing
PD: *De novo* assembly of the partial data set
PDR: Partial *de novo*-reference assembly
QC: Quality control
RiHRURs: Reads included in HRURs
RRs: Raw reads
SR: Reference assembly of the full data set with Sanger generated reference sequences
URs: Unique representative reads
