# Supplementary material for: De novo assembly of highly polymorphic metagenomic data using in situ generated reference sequences and a novel BLAST-based assembly pipeline
Source: BMC Bioinformatics. 2017 Apr 26;18:223. doi: 10.1186/s12859-017-1630-z (PMC5406902; doi:10.1186/s12859-017-1630-z)
Supplement: Supplementary file 1 — Statistics of next generation sequencing data set of HBV genome from patient serum. Table S2: Parameters used for de novo, reference, and partial de novo reference BBAP assembly. Table S3: Assembly results of individual data sets using BBAP with multiple approaches. Table S4: Comparison of polymorphism between non-overlapping and overlapping regions of D2_1 assembled contigs alignment. Table S5: Comparison of polymorphism levels between assembly results of BBAP PDR and SR assemblies. Table S6: Summary of assembled contigs from the PDR assembly of D2_1 NGS data set. Table S7: Top ten non-synonymous frequency positions of the HBV quasispecies. Table S8: Nucleotide frequencies derived from BBAP PDR assembly and pyrosequencing. Table S9: Results of BBAP de novo assembled in silico NCBI HBV complete genome (NC_003977) data sets (n = 5). Table S10: Results of Velvet assembled in silico NCBI HBV complete genome (NC_003977) data sets (n = 5). Table S11: Results of MetaVelvet assembled in silico NCBI HBV complete genome (NC_003977) data sets (n = 5). Table S12: Results of SOAPdenovo assembled in silico NCBI HBV complete genome (NC_003977) data sets (n = 5). Table S13: Results of Genovo assembled in silico NCBI HBV complete genome (NC_003977) data sets (n = 5). Table S14: Assembly time required for in silico data sets by BBAP, Velvet, MetaVelvet, SOAPdenovo, and Genovo. Table S15: Summary of study subjects and samples. Table S16: Summary of assembly results for D2_1 partial data sets of different size ratio. (DOC 461 kb) [file 12859_2017_1630_MOESM1_ESM.doc]

| Table S1. Statistics of next generation sequencing data set of HBV genome from patient serum | | | | | |
| --- | --- | --- | --- | --- | --- |
| ID | RRs | HQRs^1^ | URs | HRURs^2^ | RiHRURs |
| D2_1 | 25,232,036 | 17,264,625 | 818,554 | 72,819 | 16,324,626 |
| D2_2 | 28,321,560 | 19,393,376 | 1,272,836 | 94,547 | 17,868,725 |
| GD2_1 | 22,590,770 | 15,400,583 | 979,261 | 75,402 | 14,226,568 |
| GD2_2 | 22,444,862 | 14,724,292 | 715,088 | 41,762 | 13,916,990 |
| D3_1 | 22,182,802 | 14,666,817 | 839,898 | 49,375 | 13,703,815 |
| D3_2 | 22,750,886 | 14,928,094 | 906,127 | 66,711 | 13,851,121 |
| GD3_1 | 22,496,488 | 14,895,509 | 1,539,342 | 72,693 | 13,123,922 |
| GD3_2 | 19,747,532 | 13,581,519 | 675,328 | 40,485 | 12,785,764 |
| D1_1 | 18,734,418 | 12,415,053 | 498,452 | 14,479 | 11,815,355 |
| D1_2 | 19,744,796 | 13,090,671 | 584,253 | 70,420 | 12,423,910 |
| S1 | 14,970,060 | 9,799,111 | 513,867 | 42,912 | 9,230,382 |
| S2 | 18,715,324 | 12,506,474 | 978,725 | 81,129 | 11,389,896 |
| Average | 21,494,295 | 14,388,844 | 860,144 | 60,228 | 13,388,423 |
| RRs, raw reads; HQRs, high quality reads; URs, unique representative reads; HRURs, high redundancy unique representative reads; RiHRURs, reads included in high redundancy unique representative reads. ^1^Quality score threshold = 20, i.e., sequencing error rate = 1%; ^2^Redundancy threshold = 5. | | | | | |

| Table S2. Parameters used for *de novo*, reference, and partial *de novo* reference BBAP assembly | |
| --- | --- |
| Parameter | Value |
| Quality threshold | 20 |
| Redundancy threshold | 5 |
| BLAST e-value threshold | 1e-5 |
| BLAST identity threshold (%)^1^ | 85 |
| BLAST length threshold (bp)^1^ | 85 |
| Count per cluster threshold | 5 |
| ^1^Same identity and length threshold used for both clustering and alignment | |

| Table S3. Assembly results of individual data sets using BBAP with multiple approaches. | | | | | | |
| --- | --- | --- | --- | --- | --- | --- |
| Data set | Assembly result | FD^2^ | SR^3^ | PD^4^ | | PDR^5^ |
| D2_1 | RRs | 25,232,036 | 25,232,036 | 252,320 | 25,232,036 | |
|  | HQRs | 17,264,625 | 17,264,625 | 172,589 | 17,264,625 | |
|  | URs | 818,554 | 818,554 | 31,996 | 818,554 | |
|  | HRURs | 72,819 | 72,819 | 6,497 | 72,819 | |
|  | RiHRURs | 16,324,626 | 16,324,626 | 136,404 | 16,324,626 | |
|  | Contigs assembled^1^ | 52 | 1 | 6 | 11 | |
|  | Max contig length | 998 | 3,212 | 2,924 | 2940 | |
|  | Average contig length | 263 | 3,212 | 692 | 444 | |
|  | Mapped HRURs | 43,409 | 50,587 | 6,136 | 54,972 | |
|  | Mapped RiHRURs | 7,119,728 | 13,360,790 | 113,783 | 14,002,627 | |
| D2_2 | RRs | 28,321,560 | 28,321,560 | 283,215 | 28,321,560 | |
|  | HQRs | 19,393,376 | 19,393,376 | 194,166 | 19,393,376 | |
|  | URs | 1,272,836 | 1,272,836 | 40,986 | 1,272,836 | |
|  | HRURs | 94,547 | 94,547 | 8,205 | 94,547 | |
|  | RiHRURs | 17,868,725 | 17,868,725 | 150,135 | 17,868,725 | |
|  | Contigs assembled | 90 | 1 | 3 | 6 | |
|  | Max contig length | 1,951 | 3,212 | 3,171 | 3,202 | |
|  | Average contig length | 231 | 3,212 | 1,192 | 694 | |
|  | Mapped HRURs | 69,423 | 70,546 | 7,834 | 71,713 | |
|  | Mapped RiHRURs | 11,384,281 | 14,485,941 | 115,414 | 14,005,083 | |
| GD2_1 | RRs | 22,590,770 | 22,590,770 | 225,907 | 22,590,770 | |
|  | HQRs | 15,400,583 | 15,400,583 | 153,908 | 15,400,583 | |
|  | URs | 979,261 | 979,261 | 29,037 | 979,261 | |
|  | HRURs | 75,402 | 75,402 | 5,853 | 75,402 | |
|  | RiHRURs | 14,226,568 | 14,226,568 | 125,438 | 14,226,568 | |
|  | Contigs assembled | 52 | 1 | 1 | 3 | |
|  | Max contig length | 842 | 3,212 | 3,108 | 3,135 | |
|  | Average contig length | 240 | 3,212 | 3,108 | 1,155 | |
|  | Mapped HRURs | 52,931 | 56,348 | 5,515 | 56,086 | |
|  | Mapped RiHRURs | 7,459,016 | 12,962,670 | 86,296 | 12,959,078 | |
| GD2_2 | RRs | 22,444,862 | 22,444,862 | 224,448 | 22,444,862 | |
|  | HQRs | 14,724,292 | 14,724,292 | 147,181 | 14,724,292 | |
|  | URs | 715,088 | 715,088 | 27,207 | 715,088 | |
|  | HRURs | 41,762 | 41,762 | 7,583 | 41,762 | |
|  | RiHRURs | 13,916,990 | 13,916,990 | 117,325 | 13,916,990 | |
|  | Contigs assembled | 21 | 1 | 4 | 6 | |
|  | Max contig length | 2,531 | 3,212 | 3,116 | 3,148 | |
|  | Average contig length | 376 | 3,212 | 937 | 697 | |
|  | Mapped HRURs | 30,248 | 28,282 | 7,320 | 30,724 | |
|  | Mapped RiHRURs | 10,740,820 | 12,606,159 | 97,748 | 12,142,228 | |
| D3_1 | RRs | 22,182,802 | 22,182,802 | 221,828 | 22,182,802 | |
|  | HQRs | 14,666,817 | 14,666,817 | 146,608 | 14,666,817 | |
|  | URs | 839,898 | 839,898 | 26,405 | 839,898 | |
|  | HRURs | 49,375 | 49,375 | 7,337 | 49,375 | |
|  | RiHRURs | 13,703,815 | 13,703,815 | 121,072 | 13,703,815 | |
|  | Contigs assembled | 39 | 1 | 2 | 4 | |
|  | Max contig length | 1,610 | 3,212 | 3,087 | 3,119 | |
|  | Average contig length | 361 | 3,212 | 1,895 | 1,043 | |
|  | Mapped HRURs | 25,841 | 17,538 | 7,052 | 18,363 | |
|  | Mapped RiHRURs | 10,865,614 | 11,360,555 | 99,462 | 10,865,712 | |
| D3_2 | RRs | 22,750,886 | 22,750,886 | 227,508 | 22,750,886 | |
|  | HQRs | 14,928,094 | 14,928,094 | 149,414 | 14,928,094 | |
|  | URs | 906,127 | 906,127 | 26,648 | 906,127 | |
|  | HRURs | 66,711 | 66,711 | 6,136 | 66,711 | |
|  | RiHRURs | 13,851,121 | 13,851,121 | 124,943 | 13,851,121 | |
|  | Contigs assembled | 21 | 1 | 3 | 3 | |
|  | Max contig length | 1,318 | 3,212 | 3,180 | 3,212 | |
|  | Average contig length | 331 | 3,212 | 1,164 | 1,185 | |
|  | Mapped HRURs | 47,632 | 48,170 | 5,993 | 49,708 | |
|  | Mapped RiHRURs | 10,285,181 | 12,941,270 | 121,189 | 13,129,924 | |
| GD3_1 | RRs | 22,496,488 | 22,496,488 | 224,964 | 22,496,488 | |
|  | HQRs | 14,895,509 | 14,895,509 | 148,600 | 14,895,509 | |
|  | URs | 1,539,342 | 1,539,342 | 32,923 | 1,539,342 | |
|  | HRURs | 72,693 | 72,693 | 6,021 | 72,693 | |
|  | RiHRURs | 13,123,922 | 13,123,922 | 118,582 | 13,123,922 | |
|  | Contigs assembled | 124 | 1 | 1 | 3 | |
|  | Max contig length | 760 | 3,212 | 3,220 | 3,252 | |
|  | Average contig length | 205 | 3,212 | 3,220 | 1,192 | |
|  | Mapped HRURs | 37,391 | 28,403 | 5,743 | 30,853 | |
|  | Mapped RiHRURs | 9,104,920 | 11,540,513 | 110,070 | 11,683,987 | |
| GD3_2 | RRs | 19,747,532 | 19,747,532 | 197,475 | 19,747,532 | |
|  | HQRs | 13,581,519 | 13,581,519 | 136,023 | 13,581,519 | |
|  | URs | 675,328 | 675,328 | 20,751 | 675,328 | |
|  | HRURs | 40,485 | 40,485 | 5,655 | 40,485 | |
|  | RiHRURs | 12,785,764 | 12,785,764 | 116,147 | 12,785,764 | |
|  | Contigs assembled | 37 | 1 | 1 | 3 | |
|  | Max contig length | 1,594 | 3,212 | 3,154 | 3,183 | |
|  | Average contig length | 256 | 3,212 | 3,154 | 1,168 | |
|  | Mapped HRURs | 29,892 | 30,983 | 5,433 | 31,760 | |
|  | Mapped RiHRURs | 7,436,747 | 11,615,812 | 83,404 | 11,740,758 | |
| D1_1 | RRs | 18,734,418 | 18,734,418 | 187,344 | 18,734,418 | |
|  | HQRs | 12,415,053 | 12,415,053 | 124,271 | 12,415,053 | |
|  | URs | 498,452 | 498,452 | 13,282 | 498,452 | |
|  | HRURs | 14,479 | 14,479 | 5,681 | 14,479 | |
|  | RiHRURs | 11,815,355 | 11,815,355 | 115,413 | 11,815,355 | |
|  | Contigs assembled | 6 | 1 | 1 | 1 | |
|  | Max contig length | 1,617 | 3,208 | 3,151 | 3,180 | |
|  | Average contig length | 672 | 3,208 | 3,151 | 3,180 | |
|  | Mapped HRURs | 12,206 | 12,607 | 5,579 | 12,120 | |
|  | Mapped RiHRURs | 10,525,290 | 11,602,430 | 106,741 | 10,899,647 | |
| D1_2 | RRs | 19,744,796 | 19,744,796 | 197,447 | 19,744,796 | |
|  | HQRs | 13,090,671 | 13,090,671 | 131,044 | 13,090,671 | |
|  | URs | 584,253 | 584,253 | 22,165 | 584,253 | |
|  | HRURs | 70,420 | 70,420 | 5,832 | 70,420 | |
|  | RiHRURs | 12,423,910 | 12,423,910 | 111,129 | 12,423,910 | |
|  | Contigs assembled | 18 | 1 | 1 | 2 | |
|  | Max contig length | 1,177 | 3,208 | 3,119 | 3,147 | |
|  | Average contig length | 333 | 3,208 | 3,119 | 1,657 | |
|  | Mapped HRURs | 61,034 | 65,206 | 5,680 | 65,037 | |
|  | Mapped RiHRURs | 9,894,013 | 11,823,460 | 87,509 | 10,125,636 | |
| S1 | RRs | 14,970,060 | 14,970,060 | 149,700 | 14,970,060 | |
|  | HQRs | 9,799,111 | 9,799,111 | 97,950 | 9,799,111 | |
|  | URs | 513,867 | 513,867 | 20,085 | 513,867 | |
|  | HRURs | 42,912 | 42,912 | 4,646 | 42,912 | |
|  | RiHRURs | 9,230,382 | 9,230,382 | 76,588 | 9,230,382 | |
|  | Contigs assembled | 25 | 1 | 1 | 3 | |
|  | Max contig length | 1,999 | 3,212 | 3,086 | 3,118 | |
|  | Average contig length | 339 | 3,212 | 3,086 | 1,145 | |
|  | Mapped HRURs | 30,902 | 29,235 | 4,406 | 27,663 | |
|  | Mapped RiHRURs | 5,910,154 | 7,538,212 | 47,107 | 6,305,998 | |
| S2 | RRs | 18,715,324 | 18,715,324 | 187,153 | 18,715,324 | |
|  | HQRs | 12,506,474 | 12,506,474 | 125,194 | 12,506,474 | |
|  | URs | 978,725 | 978,725 | 34,312 | 978,725 | |
|  | HRURs | 81,129 | 81,129 | 5,727 | 81,129 | |
|  | RiHRURs | 11,389,896 | 11,389,896 | 85,484 | 11,389,896 | |
|  | Contigs assembled | 67 | 1 | 1 | 2 | |
|  | Max contig length | 1,282 | 3,212 | 3,114 | 3,141 | |
|  | Average contig length | 246 | 3,212 | 3,114 | 1,652 | |
|  | Mapped HRURs | 59,376 | 59,080 | 5,414 | 55,970 | |
|  | Mapped RiHRURs | 8,100,146 | 9,768,355 | 55,353 | 7,926,179 | |
| ^1^Minimum assembled contig length 150bp. ^2^Full data set de novo assembly; ^3^Sanger reference assembly; ^4^Partial data set de novo assembly; ^5^Partial data set reference assembly of the full data set. | | | | | | |

| Table S4. Comparison of polymorphism between non-overlapping and overlapping regions of D2_1 assembled contigs alignment | | | |
| --- | --- | --- | --- |
|  |  | Partial data set (PD) | Full data set (FD) |
| Complete region | Length | 3,213 | 2,712 |
|  | Polymorphic site | 68 | 280 |
|  | Ratio | 0.021 | 0.103 |
| Non-overlapping region | Length | 2,883 | 1,692 |
|  | Polymorphic site | 40 | 37 |
|  | Ratio | 0.014 | 0.022 |
| Overlapping region | Length | 330 | 1,020 |
|  | Polymorphic site | 28 | 243 |
|  | Ratio | 0.085^***^ | 0.238^***^ |
| ^***^p-value < 10^-10^ (Fisher’s exact test), comparison between overlapping and non-overlapping assembled regions showing in Fig. 2a. | | | |

| Table S5. Comparison of polymorphism levels between assembly results of BBAP PDR and SR assemblies. | | | | | | | | | | | | | |
| --- | --- | --- | --- | --- | --- | --- | --- | --- | --- | --- | --- | --- | --- |
| Data set | Contig length | | Overlapped length | Number of SNP sites under different SNP thresholds | | | | | | | | | |
|  |  |  |  | 0.2 | | 0.1 | | 0.01 | | 0.001 | | 0.0001 | |
|  | PDR | SR |  | PDR | SR | PDR | SR | PDR | SR | PDR | SR | PDR | SR |
| D2_1^1^ | 2,941 | 3,213 | 2,914 | 26 | 27 | 42 | 56 | 137 | 172 | 468 | 502 | 975 | 1,016 |
| D2_2 | 3,203 | 3,213 | 3,116 | 46 | 53 | 52 | 63 | 120 | 139 | 507 | 539 | 1,436 | 1,460 |
| GD2_1 | 3,136 | 3,213 | 3,109 | 6 | 5 | 13 | 14 | 123 | 142 | 599 | 622 | 1,317 | 1,334 |
| GD2_2 | 3,149 | 3,213 | 3,135 | 22 | 23 | 47 | 54 | 97 | 108 | 154 | 175 | 317 | 363 |
| D3_1 | 3,120 | 3,204 | 3,119 | 10 | 10 | 30 | 28 | 50 | 40 | 81 | 88 | 220 | 267 |
| D3_2 | 3,213 | 3,213 | 3,188 | 4 | 4 | 17 | 22 | 91 | 109 | 424 | 441 | 987 | 1,003 |
| GD3_1 | 3,253 | 3,213 | 3,200 | 1 | 6 | 4 | 15 | 58 | 80 | 490 | 506 | 1,500 | 1,500 |
| GD3_2 | 3,184 | 3,195 | 3,170 | 13 | 14 | 19 | 26 | 84 | 87 | 387 | 394 | 1,038 | 1,044 |
| D1_1 | 3,181 | 3,213 | 3,177 | 1 | 1 | 8 | 8 | 18 | 18 | 41 | 58 | 247 | 312 |
| D1_2 | 3,148 | 3,196 | 3,147 | 9 | 9 | 11 | 12 | 73 | 73 | 401 | 413 | 640 | 725 |
| S1 | 3,119 | 3,194 | 3,118 | 11 | 14 | 24 | 26 | 111 | 127 | 206 | 219 | 370 | 410 |
| S2 | 3,142 | 3,213 | 3,141 | 49 | 49 | 75 | 73 | 191 | 175 | 593 | 587 | 1,084 | 1,130 |
| ^1^D2_1 PDR assembly resulted in 2 main contigs. The longer contig was compared against SR assembly results. | | | | | | | | | | | | | |

| Table S6 Summary of assembled contigs from the PDR assembly of D2_1 NGS data set | | | | | |
| --- | --- | --- | --- | --- | --- |
| Contig | Length (bp) | HRURs | RiHRURs | Depth | Variation |
| M1 | 2,940 | 40,709 | 9,219,696 | 316,731X | n/a |
| M2 | 402 | 9,687 | 4,067,941 | 1,022,045X | n/a |
| R1 | 171 | 2,429 | 349,275 | 206,297X^1^ | 32mer quadruple repeat |
| R2 | 155 | 294 | 73,483 | 47,882X | 19mer double repeat |
| T1 | 184 | 316 | 64,805 | 35,572X | translocation |
| T2 | 180 | 259 | 36,977 | 20,748X | translocation |
| T3 | 179 | 246 | 38,453 | 21,697X | translocation |
| T4 | 179 | 372 | 58,179 | 32,827X | translocation |
| T5 | 163 | 300 | 58,219 | 36,074X | translocation |
| T6 | 162 | 221 | 19,370 | 12,076X | translocation |
| H1 | 176 | 139 | 16,229 | 9,313X | human sequence |
| ^1^The maximum number of identical 32bp duplicate copies detectable is four (see Supplementary A for detail). | | | | | |

| Table S7 Top ten non-synonymous frequency positions of the HBV quasispecies | | | | | | | |
| --- | --- | --- | --- | --- | --- | --- | --- |
| Position | Major nucleotide | Minor nucleotide | Gene | Amino acid | NS  frequency | H | Depth |
| 1,896 | G | A | C | W28Stop | 0.480 | 0.499 | 3,087,029X |
| 1,123 | C | A/T | P | R678S, R678C | 0.411 | 0.553 | 281,530X |
| 1,630 | A | T | X | H86L | 0.307 | 0.426 | 960,463X |
| 482 | C | A | P | T464N | 0.276 | 0.405 | 267,806X |
|  |  |  | S | L110I |  |  |  |
| 2,712 | G | T | P | D136Y | 0.269 | 0.394 | 220,793X |
| 167 | A | G | P | H359R | 0.249 | 0.376 | 147,227X |
|  |  |  | S | T5A |  |  |  |
| 3,088 | A | G | P | D261G | 0.214 | 0.336 | 158,801X |
|  |  |  | S1 | T81A |  |  |  |
| 1,937 | A | G | C | M13V | 0.205 | 0.328 | 535,561X |
| 2,525 | A | T | P | L73N | 0.205 | 0.326 | 279,072X |
| 2,198 | A | T | C | I100L | 0.205 | 0.325 | 441,142X |
| NS, non-synonymous; H, heterozygosity. | | | | | | | |

| Table S8. Nucleotide frequencies derived from BBAP PDR assembly and pyrosequencing | | | | | | | | | |
| --- | --- | --- | --- | --- | --- | --- | --- | --- | --- |
| Data set | Position | by BBAP PDR assembly | | | | by pyrosequencing | | | |
|  |  | A | T | C | G | A | T | C | G |
| D2_1 | 2712^1^ | - | 0.27 | - | 0.73 | - | 0.27 | - | 0.73 |
| D2_2 | 2712 | - | 0.48 | - | 0.52 | - | 0.52 | - | 0.48 |
| D2_1 | 2717 | 0.91 | - | - | 0.09 | 0.94 | - | - | 0.06 |
| D2_2 | 2717 | 0.63 | - | - | 0.37 | 0.67 | - | - | 0.33 |
| ^1^Position 2712 had the highest minor nucleotide frequency among all assembly results. | | | | | | | | | |

| Table S9. Results of BBAP *de novo* assembled *in silico* NCBI HBV complete genome (NC_003977) data sets (n=5) | | | | | | | | | | | | | |
| --- | --- | --- | --- | --- | --- | --- | --- | --- | --- | --- | --- | --- | --- |
|  |  | Error rate = 0.0001 | | | | Error rate = 0.001 | | | | Error rate = 0.01 | | | |
| Data set size |  | Mean | Std | High | Low | Mean | Std | High | Low | Mean | Std | High | Low |
| 55X | Average contig length | 3,310 | 12 | 3,324 | 3,295 | 3,321 | 14 | 3,344 | 3,310 | 2,716 | 596 | 3,312 | 1,924 |
|  | Max contig length | 3,310 | 12 | 3,324 | 3,295 | 3,321 | 14 | 3,344 | 3,310 | 3,314 | 12 | 3,332 | 3,299 |
|  | Coverage length | 3,215 | 0 | 3,215 | 3,215 | 3,215 | 0 | 3,215 | 3,215 | 3,215 | 0 | 3,215 | 3215 |
|  | Accuracy | 1 | 0 | 1 | 1 | 1 | 0 | 1 | 1 | 0.9999 | 0.0001 | 1 | 0.9997 |
|  | Number of contigs | 1 | 0 | 1 | 1 | 1 | 0 | 1 | 1 | 2 | 1 | 4 | 1 |
| 557X | Average contig length | 3,331 | 23 | 3,370 | 3,314 | 3,333 | 15 | 3,358 | 3,319 | 2,828 | 543 | 3,382 | 2,039 |
|  | Max contig length | 3,331 | 23 | 3,370 | 3,314 | 3,333 | 15 | 3,358 | 3,319 | 3,364 | 17 | 3,382 | 3,346 |
|  | Coverage length | 3,215 | 0 | 3,215 | 3,215 | 3,215 | 0 | 3,215 | 3,215 | 3,215 | 0 | 3,215 | 3215 |
|  | Accuracy | 1 | 0 | 1 | 1 | 1 | 0 | 1 | 1 | 1 | 0 | 1 | 1 |
|  | Number of contigs | 1 | 0 | 1 | 1 | 1 | 0 | 1 | 1 | 2 | 1 | 3 | 1 |
| 5,579X | Average contig length | 3,335 | 12 | 3,349 | 3,319 | 3,341 | 11 | 3,360 | 3,332 | 3,331 | 16 | 3,355 | 3,315 |
|  | Max contig length | 3,335 | 12 | 3,349 | 3,319 | 3,341 | 11 | 3,360 | 3,332 | 3,331 | 16 | 3,355 | 3,315 |
|  | Coverage length | 3,215 | 0 | 3,215 | 3,215 | 3,215 | 0 | 3,215 | 3,215 | 3,215 | 0 | 3,215 | 3215 |
|  | Accuracy | 1 | 0 | 1 | 1 | 1 | 0 | 1 | 1 | 1 | 0 | 1 | 1 |
|  | Number of contigs | 1 | 0 | 1 | 1 | 1 | 0 | 1 | 1 | 1 | 0 | 1 | 1 |
| 55,799X | Average contig length | 3,332 | 12 | 3,343 | 3,318 | 3,323 | 7 | 3,333 | 3,315 | 3,362 | 18 | 3,380 | 3,336 |
|  | Max contig length | 3,332 | 12 | 3,343 | 3,318 | 3,323 | 7 | 3,333 | 3,315 | 3,362 | 18 | 3,380 | 3,336 |
|  | Coverage length | 3,215 | 0 | 3,215 | 3,215 | 3,215 | 0 | 3,215 | 3,215 | 3,215 | 0 | 3,215 | 3215 |
|  | Accuracy | 1 | 0 | 1 | 1 | 1 | 0 | 1 | 1 | 1 | 0 | 1 | 1 |
|  | Number of contigs | 1 | 0 | 1 | 1 | 1 | 0 | 1 | 1 | 1 | 0 | 1 | 1 |

| Table S10. Results of Velvet assembled *in silico* NCBI HBV complete genome (NC_003977) data sets (n=5) | | | | | | | | | | | | | |
| --- | --- | --- | --- | --- | --- | --- | --- | --- | --- | --- | --- | --- | --- |
|  |  | Error rate = 0.0001 | | | | Error rate = 0.001 | | | | Error rate = 0.01 | | | |
| Data set size |  | Mean | Std | High | Low | Mean | Std | High | Low | Mean | Std | High | Low |
| 55X | Average contig length | 3,271 | 0 | 3,271 | 3,271 | 3,271 | 0 | 3,271 | 3,271 | 3,214 | 0 | 3,214 | 3,214 |
|  | Max contig length | 3,271 | 0 | 3,271 | 3,271 | 3,271 | 0 | 3,271 | 3,271 | 3,214 | 0 | 3,214 | 3,214 |
|  | Coverage length | 3,215 | 0 | 3,215 | 3,215 | 3,215 | 0 | 3,215 | 3,215 | 3,214 | 0 | 3,214 | 3,214 |
|  | Accuracy | 1 | 0 | 1 | 1 | 1 | 0 | 1 | 1 | 1 | 0 | 1 | 1 |
|  | Number of contigs | 1 | 0 | 1 | 1 | 1 | 0 | 1 | 1 | 1 | 0 | 1 | 1 |
| 557X | Average contig length | 3,271 | 0 | 3,271 | 3,271 | 3,211 | 6 | 3,214 | 3,201 | 429 | 62 | 488 | 326 |
|  | Max contig length | 3,271 | 0 | 3,271 | 3,271 | 3,211 | 6 | 3,214 | 3,201 | 1,087 | 465 | 1,756 | 722 |
|  | Coverage length | 3,215 | 0 | 3,215 | 3,215 | 3,211 | 6 | 3,214 | 3,201 | 3,125 | 49 | 3,184 | 3,065 |
|  | Accuracy | 1 | 0 | 1 | 1 | 1 | 0 | 1 | 1 | 0.9997 | 0.0003 | 1 | 0.9993 |
|  | Number of contigs | 1 | 0 | 1 | 1 | 1 | 0 | 1 | 1 | 9 | 2 | 12 | 7 |
| 5,579X | Average contig length | 3,208 | 13 | 3,214 | 3,185 | 604 | 112 | 696 | 411 | 95 | 87 | 162 | 0 |
|  | Max contig length | 3,208 | 13 | 3,214 | 3,185 | 1,734 | 588 | 2,548 | 1,038 | 95 | 87 | 162 | 0 |
|  | Coverage length | 3,208 | 13 | 3,214 | 3,185 | 3,086 | 80 | 3,157 | 2,993 | 93 | 85 | 162 | 0 |
|  | Accuracy | 1 | 0 | 1 | 1 | 1 | 0 | 1 | 1 | 0.5910 | 0.5395 | 0.9874 | 0 |
|  | Number of contigs | 1 | 0 | 1 | 1 | 6 | 1 | 8 | 5 | 1 | 1 | 1 | 0 |
| 55,799X | Average contig length | 1,999 | 1,156 | 3,161 | 468 | 0 | 0 | 0 | 0 | 155 | 4 | 161 | 150 |
|  | Max contig length | 2,340 | 865 | 3,161 | 1,085 | 0 | 0 | 0 | 0 | 158 | 5 | 164 | 150 |
|  | Coverage length | 3,160 | 3 | 3,164 | 3,158 | 0 | 0 | 0 | 0 | 341 | 169 | 467 | 150 |
|  | Accuracy | 1 | 0 | 1 | 1 | 0 | 0 | 0 | 0 | 0.9699 | 0.0023 | 0.9722 | 0.9667 |
|  | Number of contigs | 3 | 3 | 8 | 1 | 0 | 0 | 0 | 0 | 2 | 1 | 3 | 1 |

| Table S11. Results of MetaVelvet assembled *in silico* NCBI HBV complete genome (NC_003977) data sets (n=5) | | | | | | | | | | | | | |
| --- | --- | --- | --- | --- | --- | --- | --- | --- | --- | --- | --- | --- | --- |
|  |  | Error rate = 0.0001 | | | | Error rate = 0.001 | | | | Error rate = 0.01 | | | |
| Data set size |  | Mean | Std | High | Low | Mean | Std | High | Low | Mean | Std | High | Low |
| 55X | Average contig length | 3,271 | 0 | 3,271 | 3,271 | 3,271 | 0 | 3,271 | 3,271 | 3,214 | 0 | 3,214 | 3,214 |
|  | Max contig length | 3,271 | 0 | 3,271 | 3,271 | 3,271 | 0 | 3,271 | 3,271 | 3,214 | 0 | 3,214 | 3,214 |
|  | Coverage length | 3,215 | 0 | 3,215 | 3,215 | 3,215 | 0 | 3,215 | 3,215 | 3,214 | 0 | 3,214 | 3,214 |
|  | Accuracy | 1 | 0 | 1 | 1 | 1 | 0 | 1 | 1 | 1 | 0 | 1 | 1 |
|  | Number of contigs | 1 | 0 | 1 | 1 | 1 | 0 | 1 | 1 | 1 | 0 | 1 | 1 |
| 557X | Average contig length | 3,271 | 0 | 3,271 | 3,271 | 3,211 | 6 | 3,214 | 3,201 | 429 | 62 | 488 | 326 |
|  | Max contig length | 3,271 | 0 | 3,271 | 3,271 | 3,211 | 6 | 3,214 | 3,201 | 1,087 | 465 | 1,756 | 722 |
|  | Coverage length | 3,215 | 0 | 3,215 | 3,215 | 3,211 | 6 | 3,214 | 3,201 | 3,125 | 49 | 3,184 | 3,065 |
|  | Accuracy | 1 | 0 | 1 | 1 | 1 | 0 | 1 | 1 | 0.9997 | 0.0003 | 1 | 0.9993 |
|  | Number of contigs | 1 | 0 | 1 | 1 | 1 | 0 | 1 | 1 | 9 | 2 | 12 | 7 |
| 5,579X | Average contig length | 3,208 | 13 | 3,214 | 3,185 | 604 | 112 | 696 | 411 | 95 | 87 | 162 | 0 |
|  | Max contig length | 3,208 | 13 | 3,214 | 3,185 | 1,734 | 588 | 2,548 | 1,038 | 95 | 87 | 162 | 0 |
|  | Coverage length | 3,208 | 13 | 3,214 | 3,185 | 3,086 | 80 | 3,157 | 2,993 | 93 | 85 | 162 | 0 |
|  | Accuracy | 1 | 0 | 1 | 1 | 1 | 0 | 1 | 1 | 0.5910 | 0.5395 | 0.9874 | 0 |
|  | Number of contigs | 1 | 0 | 1 | 1 | 6 | 1 | 8 | 5 | 1 | 1 | 1 | 0 |
| 55,799X | Average contig length | 1,999 | 1,156 | 3,161 | 468 | 0 | 0 | 0 | 0 | 155 | 4 | 161 | 150 |
|  | Max contig length | 2,340 | 865 | 3,161 | 1,085 | 0 | 0 | 0 | 0 | 158 | 5 | 164 | 150 |
|  | Coverage length | 3,160 | 3 | 3,164 | 3,158 | 0 | 0 | 0 | 0 | 341 | 169 | 467 | 150 |
|  | Accuracy | 1 | 0 | 1 | 1 | 0 | 0 | 0 | 0 | 0.9699 | 0.0023 | 0.9722 | 0.9667 |
|  | Number of contigs | 3 | 3 | 8 | 1 | 0 | 0 | 0 | 0 | 2 | 1 | 3 | 1 |

| Table S12. Results of SOAPdenovo assembled *in silico* NCBI HBV complete genome (NC_003977) data sets (n=5) | | | | | | | | | | | | | |
| --- | --- | --- | --- | --- | --- | --- | --- | --- | --- | --- | --- | --- | --- |
|  |  | Error rate = 0.0001 | | | | Error rate = 0.001 | | | | Error rate = 0.01 | | | |
| Data set size |  | Mean | Std | High | Low | Mean | Std | High | Low | Mean | Std | High | Low |
| 55X | Average contig length | 0 | 0 | 0 | 0 | 0 | 0 | 0 | 0 | 3,278 | 0 | 3,278 | 3,278 |
|  | Max contig length | 0 | 0 | 0 | 0 | 0 | 0 | 0 | 0 | 3,278 | 0 | 3,278 | 3,278 |
|  | Coverage length | 0 | 0 | 0 | 0 | 0 | 0 | 0 | 0 | 3,215 | 0 | 3,215 | 3,215 |
|  | Accuracy | 0 | 0 | 0 | 0 | 0 | 0 | 0 | 0 | 1 | 0 | 1 | 1 |
|  | Number of contigs | 0 | 0 | 0 | 0 | 0 | 0 | 0 | 0 | 1 | 0 | 1 | 1 |
| 557X | Average contig length | 0 | 0 | 0 | 0 | 3,278 | 0 | 3,278 | 3,278 | 202 | 23 | 231 | 170 |
|  | Max contig length | 0 | 0 | 0 | 0 | 3,278 | 0 | 3,278 | 3,278 | 251 | 42 | 318 | 202 |
|  | Coverage length | 0 | 0 | 0 | 0 | 3,215 | 0 | 3,215 | 3,215 | 871 | 172 | 1,115 | 637 |
|  | Accuracy | 0 | 0 | 0 | 0 | 0.9999 | 0.0001 | 1 | 0.9997 | 0.9976 | 0.0019 | 1 | 0.9953 |
|  | Number of contigs | 0 | 0 | 0 | 0 | 1 | 0 | 1 | 1 | 5 | 2 | 7 | 3 |
| 5,579X | Average contig length | 3,240 | 35 | 3,278 | 3,214 | 32 | 71 | 158 | 0 | 166 | 5 | 173 | 160 |
|  | Max contig length | 3,240 | 35 | 3,278 | 3,214 | 32 | 71 | 158 | 0 | 181 | 8 | 189 | 169 |
|  | Coverage length | 3,214 | 1 | 3,215 | 3,214 | 32 | 71 | 158 | 0 | 1,393 | 445 | 1,945 | 842 |
|  | Accuracy | 1 | 0 | 1 | 1 | 0.1975 | 0.4415 | 0.9873 | 0 | 0.9881 | 0.0004 | 0.9884 | 0.9876 |
|  | Number of contigs | 1 | 0 | 1 | 1 | 0 | 0 | 1 | 0 | 11 | 4 | 14 | 5 |
| 55,799X | Average contig length | 73 | 100 | 192 | 0 | 0 | 0 | 0 | 0 | 134 | 76 | 184 | 0 |
|  | Max contig length | 73 | 100 | 192 | 0 | 0 | 0 | 0 | 0 | 134 | 76 | 184 | 0 |
|  | Coverage length | 73 | 100 | 192 | 0 | 0 | 0 | 0 | 0 | 134 | 76 | 184 | 0 |
|  | Accuracy | 0.3956 | 0.5417 | 0.9896 | 0 | 0 | 0 | 0 | 0 | 0.7797 | 0.4359 | 0.9759 | 0 |
|  | Number of contigs | 0 | 1 | 1 | 0 | 0 | 0 | 0 | 0 | 1 | 0 | 1 | 0 |

| Table S13. Results of Genovo assembled *in silico* NCBI HBV complete genome (NC_003977) data sets (n=5) | | | | | | | | | | | | | |
| --- | --- | --- | --- | --- | --- | --- | --- | --- | --- | --- | --- | --- | --- |
|  |  | Error rate = 0.0001 | | | | Error rate = 0.001 | | | | Error rate = 0.01 | | | |
| Data set size |  | Mean | Std | High | Low | Mean | Std | High | Low | Mean | Std | High | Low |
| 55X | Average contig length | 3,309 | 2 | 3,311 | 3,306 | 3,309 | 3 | 3,312 | 3,306 | 3,310 | 3 | 3,315 | 3,307 |
|  | Max contig length | 3,309 | 2 | 3,311 | 3,306 | 3,309 | 3 | 3,312 | 3,306 | 3,310 | 3 | 3,315 | 3,307 |
|  | Coverage length | 3,215 | 0 | 3,215 | 3,215 | 3,215 | 0 | 3,215 | 3,215 | 3,215 | 0 | 3,215 | 3,215 |
|  | Accuracy | 1 | 0 | 1 | 1 | 1 | 0 | 1 | 1 | 1 | 0 | 1 | 1 |
|  | Number of contigs | 1 | 0 | 1 | 1 | 1 | 0 | 1 | 1 | 1 | 0 | 1 | 1 |
| 557X | Average contig length | 3,315 | 0 | 3,316 | 3,315 | 3,316 | 1 | 3,317 | 3,315 | 3,319 | 4 | 3,324 | 3,316 |
|  | Max contig length | 3,315 | 0 | 3,316 | 3,315 | 3,316 | 1 | 3,317 | 3,315 | 3,319 | 4 | 3,324 | 3,316 |
|  | Coverage length | 3,215 | 0 | 3,215 | 3,215 | 3,215 | 0 | 3,215 | 3,215 | 3,215 | 0 | 3,215 | 3,215 |
|  | Accuracy | 1 | 0 | 1 | 1 | 1 | 0 | 1 | 1 | 1 | 0 | 1 | 1 |
|  | Number of contigs | 1 | 0 | 1 | 1 | 1 | 0 | 1 | 1 | 1 | 0 | 1 | 1 |
| 5,579X | Average contig length | 3,323 | 0 | 3,323 | 3,323 | 3,323 | 0 | 3,323 | 3,323 | 3,336 | 14 | 3,361 | 3,327 |
|  | Max contig length | 3,323 | 0 | 3,323 | 3,323 | 3,323 | 0 | 3,323 | 3,323 | 3,336 | 14 | 3,361 | 3,327 |
|  | Coverage length | 3,215 | 0 | 3,215 | 3,215 | 3,215 | 0 | 3,215 | 3,215 | 3,215 | 0 | 3,215 | 3,215 |
|  | Accuracy | 1 | 0 | 1 | 1 | 1 | 0 | 1 | 1 | 0.9999 | 0.0001 | 1 | 0.9997 |
|  | Number of contigs | 1 | 0 | 1 | 1 | 1 | 0 | 1 | 1 | 1 | 0 | 1 | 1 |
| 55,799X | Average contig length | 1,713 | 271 | 2,184 | 1,531 | 2,031 | 881 | 3,498 | 1,303 | 798 | 118 | 952 | 620 |
|  | Max contig length | 2,761 | 607 | 3,528 | 2,247 | 3,555 | 118 | 3,757 | 3,455 | 1,816 | 515 | 2,412 | 1,178 |
|  | Coverage length | 3,215 | 0 | 3,215 | 3,215 | 3,215 | 0 | 3,215 | 3,215 | 3,215 | 0 | 3,215 | 3,215 |
|  | Accuracy | 1 | 0 | 1 | 1 | 1 | 0 | 1 | 1 | 0.9990 | 0.0005 | 0.9996 | 0.9986 |
|  | Number of contigs | 3 | 0 | 3 | 2 | 2 | 1 | 3 | 1 | 5 | 1 | 7 | 4 |

| Table S14. Assembly time required for *in silico* data sets by BBAP, Velvet, MetaVelvet, SOAPdenovo, and Genovo | | | | | | | |
| --- | --- | --- | --- | --- | --- | --- | --- |
|  |  | Assembly time (sec) | | | | | |
|  |  | Error rate = 0.0001 | | Error rate = 0.001 | | Error rate = 0.01 | |
| Data set size |  | avg | std | avg | std | avg | std |
| 55X | BBAP | 28.4 | 0.9 | 29.8 | 0.8 | 40.4 | 1.1 |
|  | Velvet | 0.2 | 0.4 | 0.4 | 0.5 | 0.2 | 0.4 |
|  | MetaVelvet | 0.2 | 0.4 | 0.2 | 0.4 | 0.2 | 0.4 |
|  | SOAPdenovo | 4.0 | 0.0 | 6.0 | 2.2 | 4.8 | 0.8 |
|  | Genovo | 1,320.0 | 42.4 | 1,380.0 | 42.4 | 1,416.0 | 32.9 |
| 557X | BBAP | 53.6 | 1.1 | 42.6 | 0.5 | 50.6 | 1.1 |
|  | Velvet | 1.0 | 0.0 | 1.4 | 0.9 | 1.6 | 0.9 |
|  | MetaVelvet | 1.2 | 0.4 | 1.4 | 0.5 | 1.8 | 0.4 |
|  | SOAPdenovo | 4.8 | 0.4 | 6.2 | 1.6 | 8.4 | 3.2 |
|  | Genovo | 5,136.0 | 144.5 | 5,304.0 | 138.1 | 5,616.0 | 68.4 |
| 5,579X | BBAP | 131.4 | 0.5 | 131.6 | 0.9 | 133.8 | 1.3 |
|  | Velvet | 8.8 | 0.8 | 12.4 | 1.1 | 25.6 | 0.9 |
|  | MetaVelvet | 12.2 | 1.9 | 16.0 | 0.7 | 63.0 | 7.3 |
|  | SOAPdenovo | 13.4 | 0.5 | 18.4 | 1.5 | 19.4 | 1.7 |
|  | Genovo | 22,620.0 | 2,752.5 | 49,596.0 | 63,607.5 | 22,548.0 | 370.8 |
| 55,799X | BBAP | 139.6 | 1.1 | 141.6 | 0.5 | 203.2 | 2.2 |
|  | Velvet | 99.8 | 6.9 | 159.2 | 5.5 | 218.2 | 0.8 |
|  | MetaVelvet | 151.6 | 4.4 | 541.2 | 55.4 | 757.8 | 58.7 |
|  | SOAPdenovo | 111.8 | 1.9 | 120.4 | 8.2 | 146.6 | 3.8 |
|  | Genovo | 65,808.0 | 35,194.3 | 49,476.0 | 270.3 | 49,776.0 | 715.7 |

| Table S15. Summary of study subjects and samples. | | | | |
| --- | --- | --- | --- | --- |
| Individual | Sample ID | Year of Birth | Year of Sampling | HBV DNA level  (log10 copies/ml) |
| Daughter 1 | D1_1 | 1944 | 2006 | 4.97 |
|  | D1_2 |  | 2009 | 5.51 |
| Daughter 2 | D2_1 | 1949 | 1997 | 6.50 |
|  | D2_2 |  | 2000 | 7.66 |
| Daughter 3 | D3_1 | 1952 | 2007 | 4.51 |
|  | D3_2 |  | 2010 | 6.33 |
| Son 1 | S1 | 1956 | 2008 | 5.01 |
| Son 2 | S2 | 1959 | 2008 | 7.20 |
| Granddaughter 2^1^ | GD2_1 | 1977 | 2004 | 5.02 |
|  | GD2_2 |  | 2006 | 7.86 |
| Granddaughter 3^1^ | GD3_1 | 1980 | 2005 | 7.43 |
|  | GD3_2 |  | 2010 | 7.22 |
| ^1^Granddaughter 2 and 3 are the daughters of Daughter 2 and 3, respectively. | | | | |

| Table S16. Summary of assembly results for D2_1 partial data sets of different size ratio. | | | | | |
| --- | --- | --- | --- | --- | --- |
| Size ratio | Mapped HRURs ratio | Mapped RiHRURs ratio | Contigs assembled | Max contig length | Average contig length |
| 40% | 0.40(0.09) | 0.27(0.07) | 6.8(0.8) | 1,402(379) | 826(228) |
| 20% | 0.55(0.12) | 0.43(0.13) | 9.2(1.6) | 1,776(658) | 546(85) |
| 10% | 0.59(0.11) | 0.51(0.13) | 5.8(1.3) | 2,063(336) | 846(252) |
| 1% | 0.97(0.00) | 0.94(0.03) | 5.8(0.8) | 2,579(409) | 696(83) |
| 0.1% | 0.62(0.13) | 0.58(0.31) | 2.6(0.5) | 190(14) | 177(12) |
| 0.01% | 0(0) | 0(0) | 0(0) | n/a | n/a |
| Assembly result data are presented in form of avg(std). Size ratio, the ratio of raw reads randomly selected from the full D2_1 data set for partial data sets. HRURs, high redundancy unique representative reads (unique representative reads with redundancy threshold = 5); RiHRURs, reads included in high redundancy unique representative reads. | | | | | |
