# Supplementary material for: De novo assembly of highly polymorphic metagenomic data using in situ generated reference sequences and a novel BLAST-based assembly pipeline
Source: BMC Bioinformatics. 2017 Apr 26;18:223. doi: 10.1186/s12859-017-1630-z (PMC5406902; doi:10.1186/s12859-017-1630-z)
Supplement: Supplementary file 2 — Comparison of HBV recover ratio by BBAP, Velvet, SOAPdenovo, and Genovo assembly of full and partial D2_1 data sets. Figure S2: Correlation between assembled scaffold length and scaffold degeneracy for all 12 data sets. Figure S3: (a) Nucleotide sequence of the R1 scaffold. (b) Schematic alignment of the R1 scaffold, HBV X gene and HBV precore/core gene. Figure S4: Schematic diagram of the T1 scaffold and its corresponding HBV genome regions. Figure S5: Schematic diagram of the T6 scaffold and its corresponding HBV genome and Sanger reference sequence regions. Figure S6: Alignment of Sanger (SR) and partial D2_1 data set assembled scaffolds reference assembled (PDR) scaffolds to the Sanger reference sequence. Figure S7: Diversity profile of D2_1 HBV quasispecies according to assembly results of partial data set reference assembly of the full data set. Figure S8: Schematic diagram of two Genovo assembled scaffolds with identified HBV structural variants and its corresponding HBV genome regions. (PDF 489 kb) [file 12859_2017_1630_MOESM2_ESM.pdf]

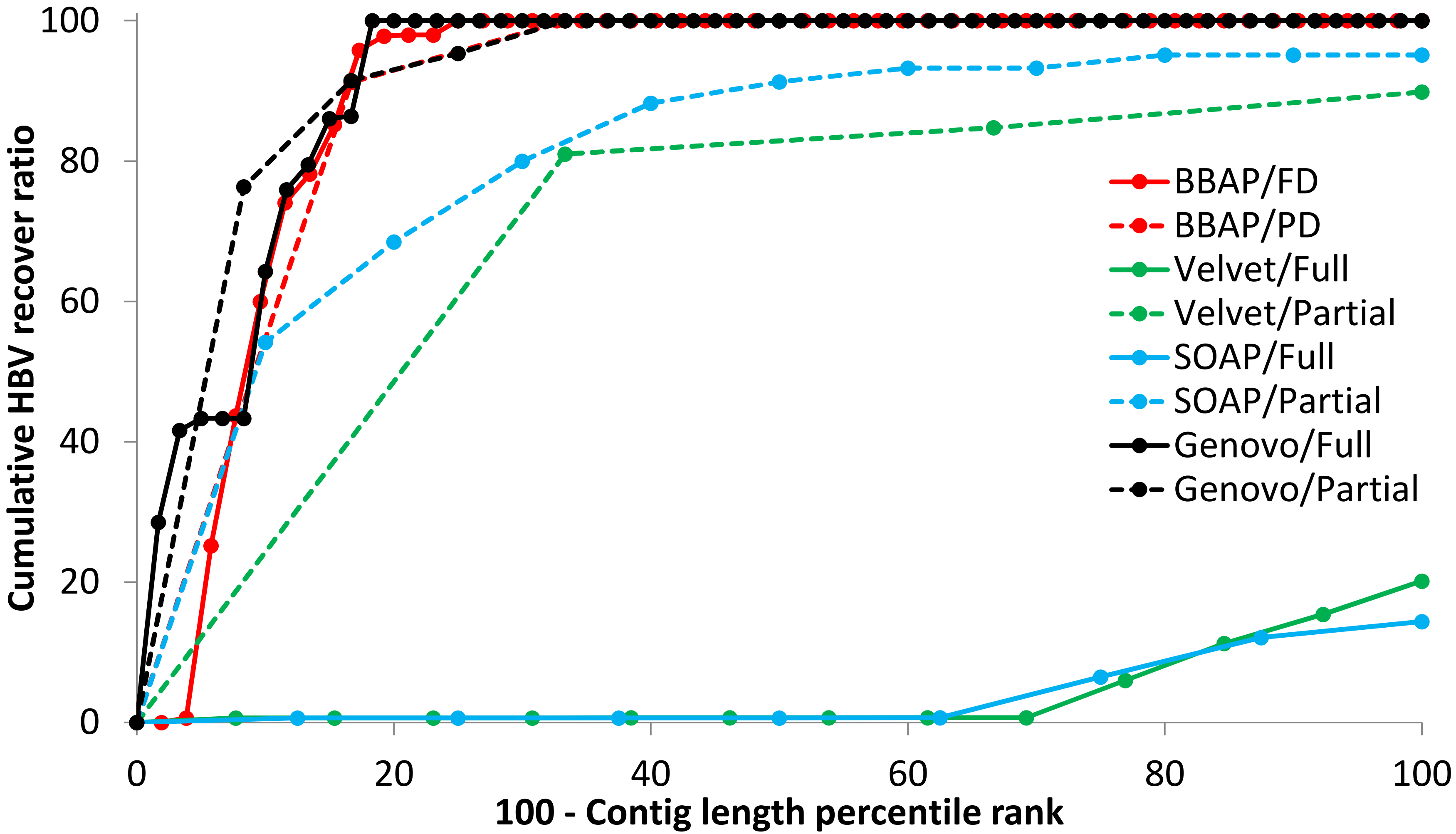

Figure S1. Comparison of HBV recover ratio by BBAP, Velvet, SOAPdenovo, and Genovo assembly of full and partial D2_1 data sets. The steeper and more rapid incline for assemblies of the partial data set compared to that of the full data sets suggest partial data set assembly results in more complete and less fragmented recovery of the reference HBV genome than full data set assembly.

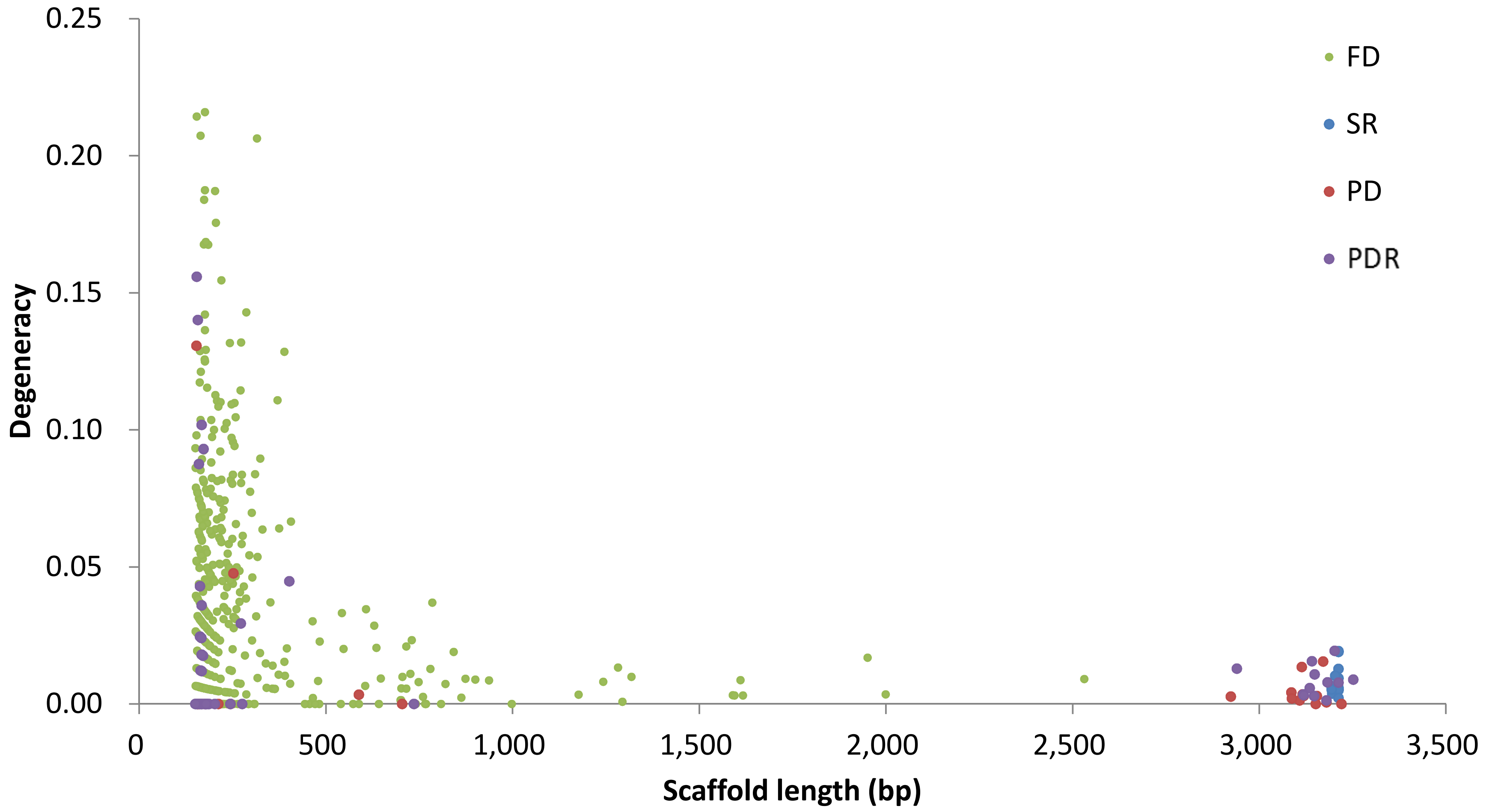

Figure S2. Correlation between assembled scaffold length and scaffold degeneracy for all 12 data sets. Degeneracy is the ratio of assembled polymorphic sites. FD assembly, full data set *de novo* assembly; SR assembly, full data set reference assembly with Sanger sequence as reference; PD assembly, partial data set *de novo* assembly; PDR assembly, full data set reference assembly with PD assembly results as reference.

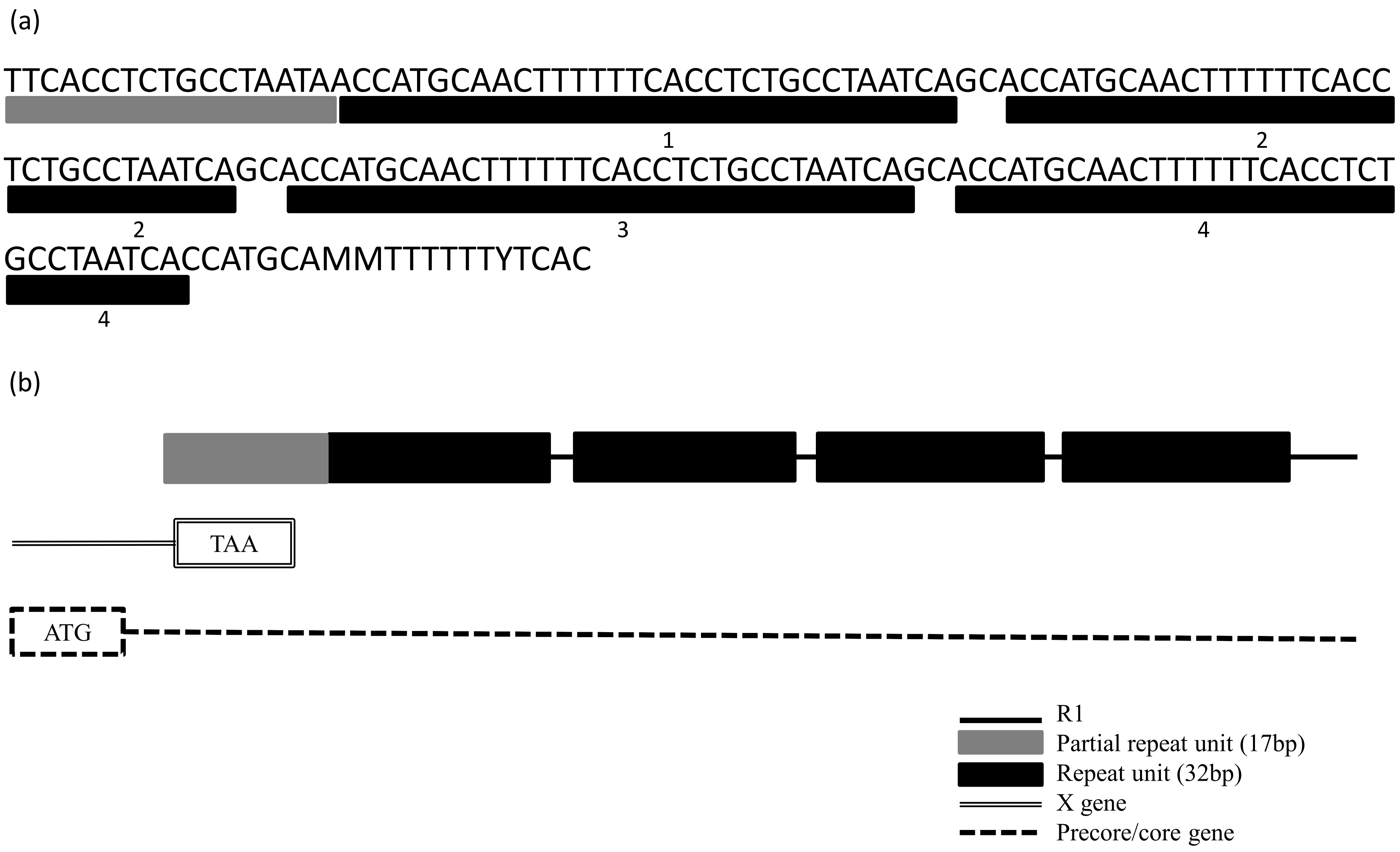

Figure S3. (a) Nucleotide sequence of the R1 scaffold. (b) Schematic alignment of the R1 scaffold, HBV X gene and HBV precore/core gene.

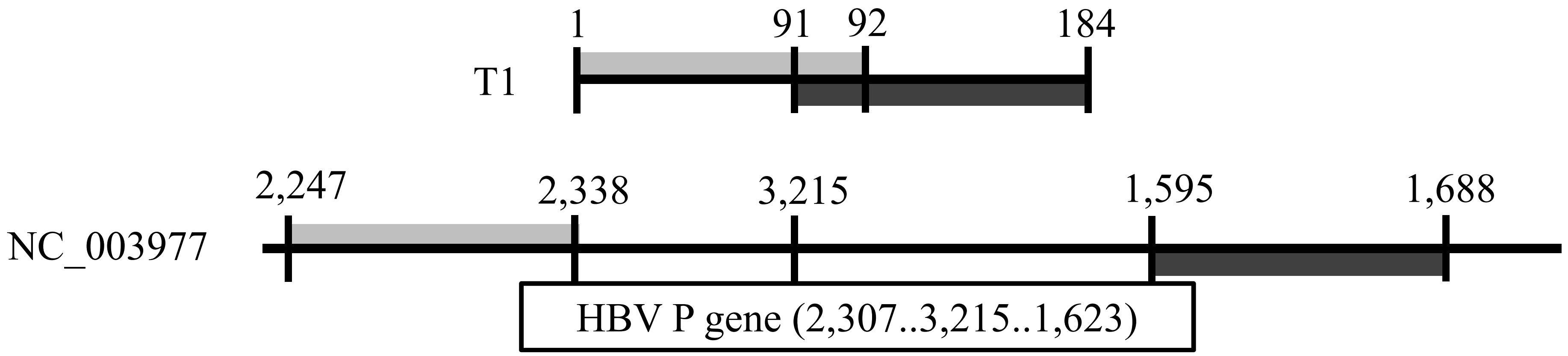

Figure S4. Schematic diagram of the T1 scaffold and its corresponding HBV genome regions.

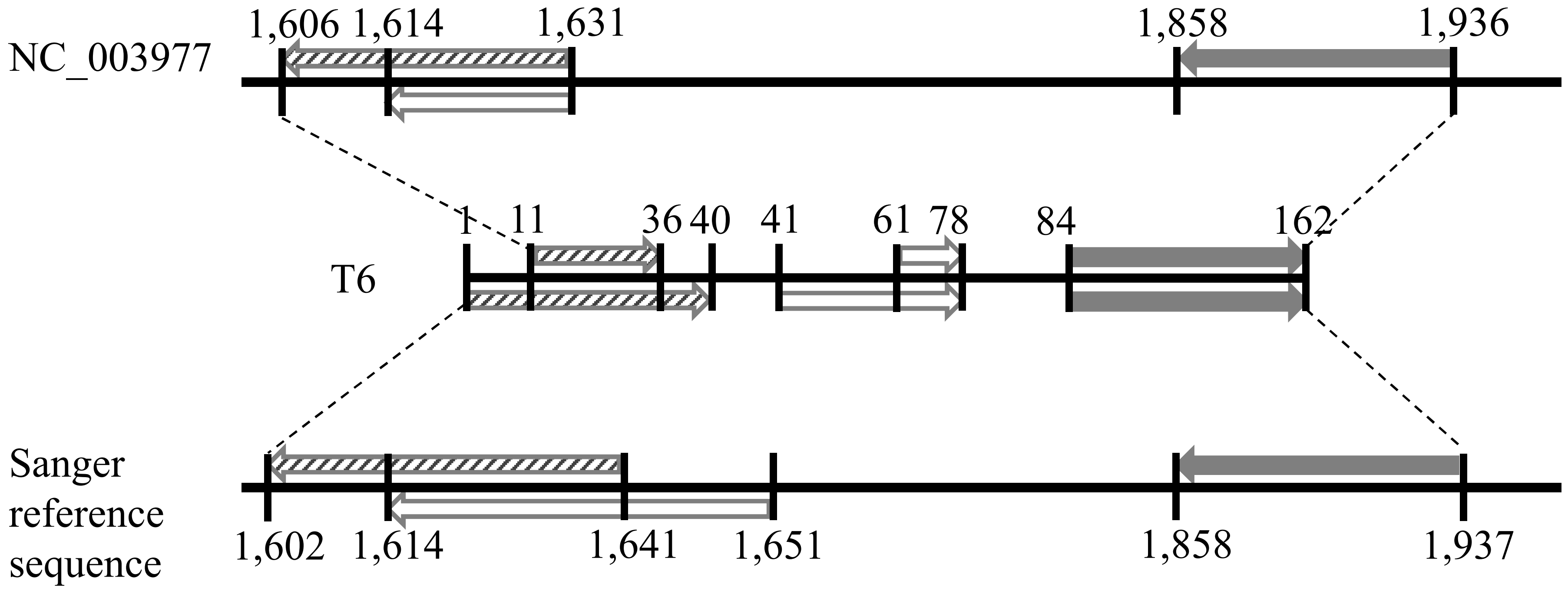

Figure S5. Schematic diagram of the T6 scaffold and its corresponding HBV genome and Sanger reference sequence regions. For the T6 scaffold, the top level indicates the matched regions to NC_003977, and the bottom level indicate regions matched to the Sanger reference sequence. The difference of regions matched between NC_003977 and the Sanger reference sequence is due to the sequence differences between the two reference sequences. Positions are indicated in reference to NC_003977.

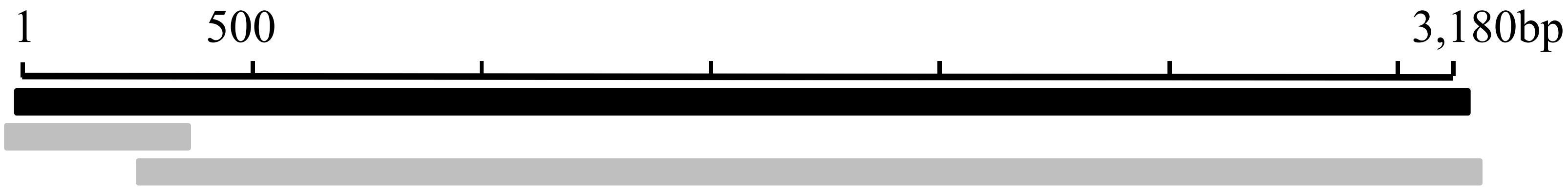

Sanger reference sequence
BBAP full data set assembled scaffolds with Sanger reference
BBAP full data set assembled scaffolds with partial data set *de novo* assembled reference

Figure S6. Alignment of Sanger (SR) and partial D2_1 data set assembled scaffolds reference assembled (PDR) scaffolds to the Sanger reference sequence.

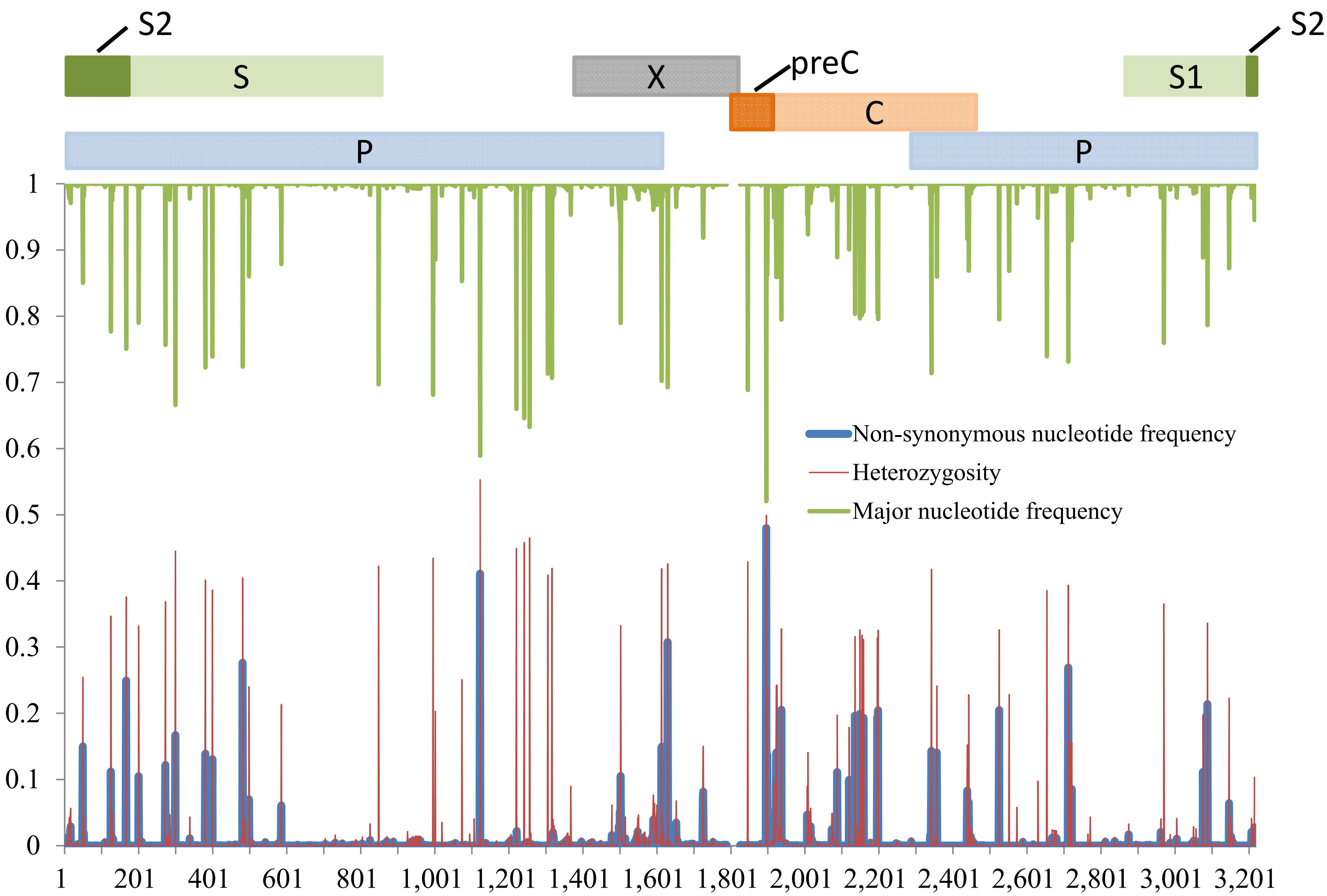

Figure S7. Diversity profile of D2_1 HBV quasispecies according to assembly results of partial data set reference assembly of the full data set. The non-synonymous nucleotide frequency is the sum of nucleotide frequencies that result in non-synonymous amino acid changes. Reference amino acids are determined by the nucleotides with the highest frequency of each position.

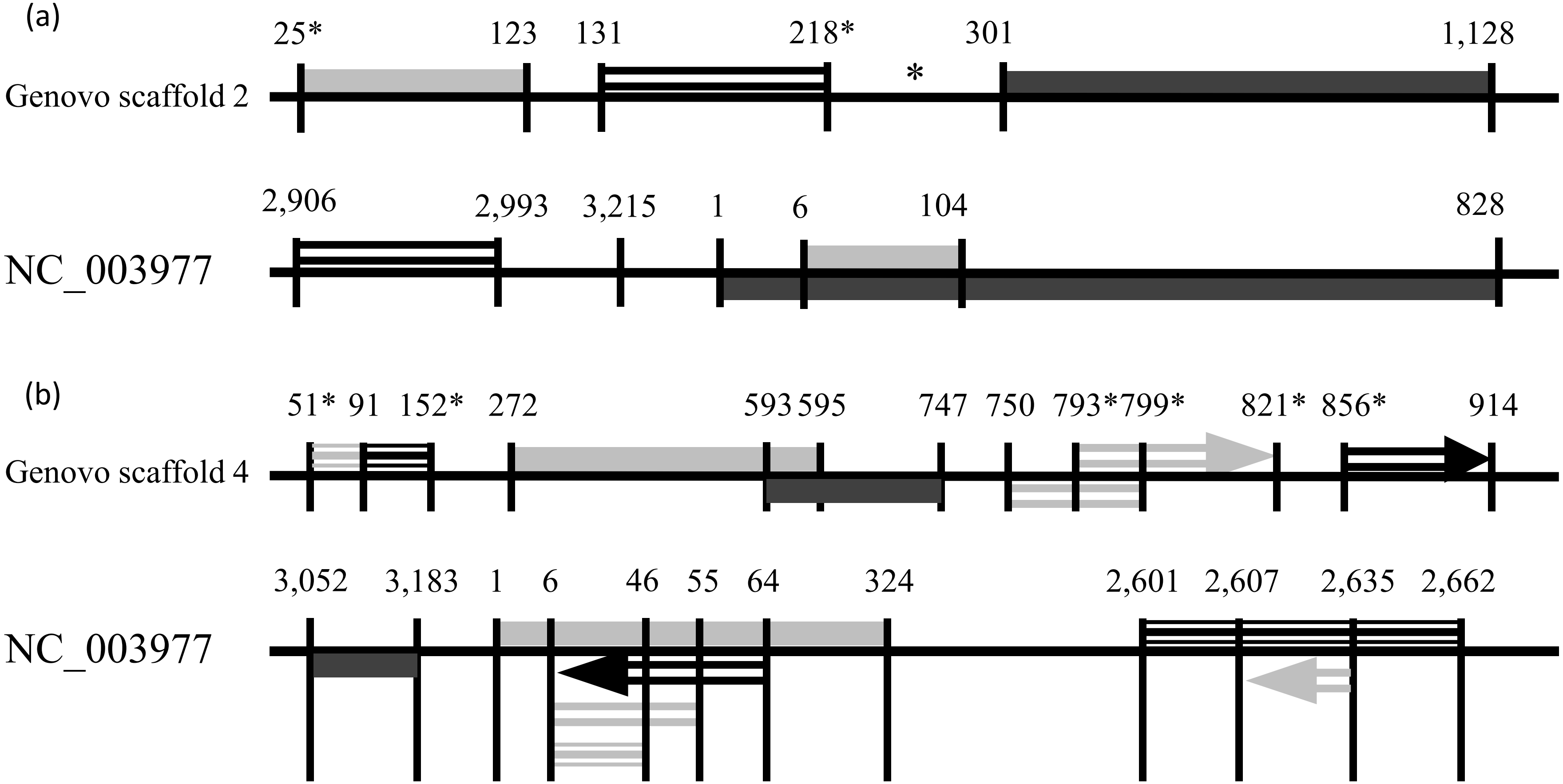

Figure S8. Schematic diagram of two Genovo assembled scaffolds with identified HBV structural variants and its corresponding HBV genome regions. Asterisk (*) marked positions or regions indicate the corresponding sequences were not found in the NGS data set. The corresponding sequence for asterisk marked positions or regions consists of the sequences 20bp downstream and upstream of the marked position or the center position of the marked region, respectively.
