# Supplementary material for: De novo assembly of highly polymorphic metagenomic data using in situ generated reference sequences and a novel BLAST-based assembly pipeline
Source: BMC Bioinformatics. 2017 Apr 26;18:223. doi: 10.1186/s12859-017-1630-z (PMC5406902; doi:10.1186/s12859-017-1630-z)
Supplement: Supplementary file 3 — A. Variant sequences and human genome sequences. B. Diversity profile of D2_1 HBV quasispecies. C. Structure variation by Genovo. D. Determining optimal size of partial data set. (DOC 67 kb) [file 12859_2017_1630_MOESM3_ESM.doc]

**Supplementary Information**

1. **Variant sequences and human genome sequences**

Of the total 47 contigs assembled from the 12 PDR assemblies, only 15 contigs were mapped to the full length HBV genome, whereas among the remaining 32 contigs, 11 were identified to be HBV structural variants including repetitive sequences, long deletion, and translocation, and the remaining 21 contigs were all partially matched to the full length HBV genome. Eight of the 11 HBV structural variants were from the D2_1 data set (Supplementary Table S6).

Two variant contigs, R1 and R2, were identified as tandem repeats. The former contig consists of a 32mer quadruple repeat (Supplementary Fig. S3), whereas the latter contig contains a 19mer double repeat. The leading 17bp region of the R1 contig, excluding a single nucleotide difference, corresponds to the latter 17bp of the 32bp repeat unit. Combined with the positioning of the leading 17bp region and the 2 nucleotide gaps found between 32mer repeats but not between the leading region and the first repeat unit, suggest the 17bp leading region is part of the duplication origin rather than an additional duplicate copy. The quadruple copies of the R1 contig 32mer repeat unit are located downstream of both the precore/core gene start codon and the X gene stop codon but upstream of the core gene start codon, which suggests alterations to the precore/core gene translation but not the X gene or core gene translations. Core initiated translation results in the production of HBcAg, whereas precore initiated translation of the C gene produces HBeAg, the extracellular form of HBcAg and not required for replication . Because the detection limitation of 32bp identical repeats using 101bp reads is four duplicate copies, it is reasonable to expect the actual number of tandem repeats is greater than the detected 4 copies, therefore the estimated R1 contig coverage depth of 206,297X is an overestimation. The 19mer R2 repeat unit was located in the non-overlapping region of the X gene.

Six variant contigs, T1-T6, were found to contain immediately adjacent or overlapping regions that correspond to distant regions in the HBV genome (Fig. 2). Such structural variation could be results of translocation recombination, gene conversion, or deletion. For example, the anterior half of the T1 contig is perfectly matched to the 5’end of the HBV polymerase gene of the D2_1 Sanger reference sequence, whereas the posterior half is a 98.9% (93/94) identity match to the 3’end of the same polymerase gene (Supplementary Fig. S4). The two overlapping halves of the T1 contig are separately matched to regions that are at least 2.4kb apart in the HBV genome. The T2, T3, T4, and T5 contigs all have similar structures as the T1 contig. The T6 contig not only has three homologous regions, but also has different synteny and orientation than the other contigs (Supplementary Fig. S5). Compared to the two main contigs, M1 and M2, the relative coverage of T1-T5 variants ranged 0.049 (T2) to 0.085 (T5), suggesting that they were not single molecule residing in the quasispecies. Further experiment also demonstrated the existence of similar variants in the population (Fig. 2). Two of the three non-overlapping T6 matching regions were found to correspond to overlapping genomic regions in both the NCBI HBV complete genome sequence and the Sanger reference sequence, suggesting the involvement of a duplication event as well.

The H1 contig (Supplementary Table S6), consisting of 0.1% of the mapped RiHRURs, was matched to chromosome 6 of the human genome. No significant HBV homology was found to suggest virus integration into the human genome. Given its relatively low coverage depth and sequence length (176bp), the H1 contig is possibly the result of residual human DNA during the extraction of virus DNA from blood serum. Similar structural variants were also found among the 51 contigs from the D2_1 FD assembly. A total of 31 contigs have homologous regions that suggest the occurrence of deletion or translocation events, and another 3 contigs included tandem repeats. A single contig was identified as human genome sequence.

To validate the assembly results of these HBV variant, PCR designed to target these regions were performed. The PCR product were extracted and sequenced. The sequence revealed a 291 bp variant contig, L1, which not only has the same synteny and orientation as the T5 variant contig, but also near identical junction positions as well (Figure 2). Interestingly, all variants (T1-T5 and L1) retained encapsidation signal (or episilon, ε) required for the packaging of pregenomic RNA. Therefore, it is possible that the translocation variants, similar to defective interfering particles, retain their ability to properly replicate, package and be secreted through defective interference .

1. **Diversity profile of D2_1 HBV quasispecies**

The D2_1 HBV quasispecies diversity profile was constructed using the M1 and M2 contigs from PDR assembly (Supplementary Fig. S7). The heterozygosity (H) of the assembled HBV genome was relatively low with an average of 0.008, whereas less than 2% of the assembled HBV genome exhibited high polymorphism (58/3180, H > 0.1). To examine sites possibly under selection, we calculated the frequency of non-synonymous nucleotides for each position. Among the top ten positions with the highest non-synonymous nucleotide frequencies, several were found to be related with hepatitis B and hepatocellular carcinoma (HCC) in recent studies (Supplementary Table S7). The polymorphic G/A of position 1,896 was found to have the highest non-synonymous frequency while representing an amino acid change from tryptophan to a premature stop codon. This G1896A substitution is commonly found in chronic hepatitis patients . It has been suggested that because the HBV core antigen is an important immunological target of the host immune system, such changes may alter the recognition sites and assist escaping host immune responses. Amino acid changes at HBx86 (position 1,630) has also been reported to possibly contribute to HCC development through activation of the NF-κB pathway in HCC patients . Additionally, mutation T2712C/A/G was found to be associated with HCC while HBc13 mutations were associated with HLA-I alleles , and position 1,123 is located in the genomic region of HLA-II alleles . Other sites also with high non-synonymous nucleotide frequency may be potential candidates for future HBV-related studies.

1. **Structure variation by Genovo**

BLAST results of the Genovo full data set assembly results against the Sanger reference sequence indicated only 3 of the 37 non-human contigs did not contain HBV structural variations. Although several Genovo identified structural variants were consistent with the BBAP identified variants in Supplementary Table S6**,** the distribution pattern of identified variants among assembled contigs were different; each Genovo assembled contigs included multiple variants, whereas each BBAP assembled contigs only included a single variant. Several junction regions between identified structural variants of the same Genovo contig were found to be non-existent in the NGS data set (Supplementary Fig. S8). Taken together, the authenticity of the Genovo identified structural variants is questionable.

1. **Determining optimal size of partial data set**

The optimal partial data set should exclude noise signals of highly polymorphic samples but remain representative of both the majority sequence and minor variants. To determine the optimal size for partial data sets in this study, we randomly extracted and assembled partial data sets of a wide range of size ratio (40%, 20%, 10%, 1%, 0.1%, and 0.01%) using the D2_1 data set. We independently and randomly extracted 5 partial data sets from the full D2_1 data set for each size ratio, followed by BBAP *de novo* assembly (Supplementary Table S16). Assembly of partial data sets with size ratio of 1% resulted in the highest ratio of mapped reads for both HRURs and RiHRURs as well as the longest average maximum contig length, suggesting 1% as the optimal size ratio to retain majority of genetic information while achieving high assembling efficiency.

To test whether if the assembly quality improvement by partial *de novo*-reference assembly was limited to the data sets analyzed in this study, we applied the partial *de novo*-reference assembly approach to other NGS data sets sequenced from various organisms, including *Escherichia coli*, *Saccharomyces cerevisiae*, multiple species of fish from the genus *Zacco*, and human (data not shown). The assembly quality of these data sets were also improved compared to the original assembly results using the respective full data sets. Additionally, the optimal size ratio for partial data sets were different among data sets. We hypothesize that the optimal size ratio is dependent upon multiple factors of the full data set, including total number of reads, coverage depth, degree of polymorphism, and NGS read length. With further analyses, we wish to develop a systematic method to predict the optimal size ratio for each specific NGS data set in future studies.

**Reference**

1. Buti M, Rodriguez-Frias F, Jardi R, Esteban R: **Hepatitis B virus genome variability and disease progression: the impact of pre-core mutants and HBV genotypes**. *Journal of clinical virology : the official publication of the Pan American Society for Clinical Virology* 2005, **34 Suppl 1**:S79-82.

2. Tacke F, Gehrke C, Luedde T, Heim A, Manns MP, Trautwein C: **Basal core promoter and precore mutations in the hepatitis B virus genome enhance replication efficacy of Lamivudine-resistant mutants**. *Journal of virology* 2004, **78**(16):8524-8535.

3. Yuan TT, Lin MH, Chen DS, Shih C: **A defective interference-like phenomenon of human hepatitis B virus in chronic carriers**. *Journal of virology* 1998, **72**(1):578-584.

4. Lee YI, Hur GM, Suh DJ, Kim SH: **Novel pre-C/C gene mutants of hepatitis B virus in chronic active hepatitis: naturally occurring escape mutants**. *The Journal of general virology* 1996, **77 ( Pt 6)**:1129-1138.

5. Parvez MK: **"Dinucleotide-pattern" GA Hypermutations in the pre-Core 5-GGGG Tetrad of HBeAg Negative Variants of Hepatitis B Virus**. *J Gastroen Hepatol Res* 2013, **2**(9).

6. Lee JH, Han KH, Lee JM, Park JH, Kim HS: **Impact of hepatitis B virus (HBV) x gene mutations on hepatocellular carcinoma development in chronic HBV infection**. *Clinical and vaccine immunology : CVI* 2011, **18**(6):914-921.

7. Sung JJ, Tsui SK, Tse CH, Ng EY, Leung KS, Lee KH, Mok TS, Bartholomeusz A, Au TC, Tsoi KK *et al*: **Genotype-specific genomic markers associated with primary hepatomas, based on complete genomic sequencing of hepatitis B virus**. *Journal of virology* 2008, **82**(7):3604-3611.

8. Abbott WG, Tsai P, Leung E, Trevarton A, Ofanoa M, Hornell J, Gane EJ, Munn SR, Rodrigo AG: **Associations between HLA class I alleles and escape mutations in the hepatitis B virus core gene in New Zealand-resident Tongans**. *Journal of virology* 2010, **84**(1):621-629.

9. Desmond CP, Bartholomeusz A, Gaudieri S, Revill PA, Lewin SR: **A systematic review of T-cell epitopes in hepatitis B virus: identification, genotypic variation and relevance to antiviral therapeutics**. *Antiviral therapy* 2008, **13**(2):161-175.
